# Proinflammatory immune cells disrupt angiogenesis and promote germinal matrix hemorrhage in prenatal human brain

**DOI:** 10.1038/s41593-024-01769-2

**Published:** 2024-09-30

**Authors:** Jiapei Chen, Elizabeth E. Crouch, Miriam E. Zawadzki, Kyle A. Jacobs, Lakyn N. Mayo, Jennifer Ja-Yoon Choi, Pin-Yeh Lin, Saba Shaikh, Jessica Tsui, Susana Gonzalez-Granero, Shamari Waller, Avani Kelekar, Gugene Kang, Edward J. Valenzuela, Janeth Ochoa Birrueta, Loukas N. Diafos, Kaylee Wedderburn-Pugh, Barbara Di Marco, Wenlong Xia, Claudia Z. Han, Nicole G. Coufal, Christopher K. Glass, Stephen P. J. Fancy, Julieta Alfonso, Arnold R. Kriegstein, Michael C. Oldham, Jose Manuel Garcia-Verdugo, Matthew L. Kutys, Maria K. Lehtinen, Alexis J. Combes, Eric J. Huang

**Affiliations:** 1 Department of Pathology and Weill Institute for Neurosciences, University of California San Francisco, San Francisco, CA, USA.; 2 Biomedical Sciences Graduate Program, University of California San Francisco, San Francisco, CA, USA.; 3 Department of Pediatrics, University of California San Francisco, San Francisco, CA, USA.; 4 The Eli and Edythe Broad Center of Regeneration Medicine and Stem Cell Research, University of California San Francisco, San Francisco, CA, USA.; 5 Department of Pathology, Boston Children’s Hospital and Harvard Medical School, Boston, MA, USA.; 6 Harvard/MIT MD–PhD Program, Harvard Medical School, Boston, MA, USA.; 7 Graduate Program in Biological and Biomedical Sciences, Harvard Medical School, Boston, MA, USA.; 8 Department of Cell and Tissue Biology, University of California San Francisco, San Francisco, CA, USA.; 9 UCSF–UC Berkeley Joint Graduate Program in Bioengineering, University of California San Francisco, San Francisco, CA, USA.; 10 UCSF CoLab, University of California San Francisco, San Francisco, CA, USA.; 11 Laboratory of Comparative Neurobiology, Cavanilles Institute of Biodiversity and Evolutionary Biology, University of Valencia and CIBERNED-ISCIII, Valencia, Spain.; 12 Department of Neurological Surgery, University of California San Francisco, San Francisco, CA, USA.; 13 Developmental and Stem Cell Biology Graduate Program, University of California, San Francisco, CA, USA.; 14 Medical Scientist Training Program, University of California San Francisco, San Francisco, CA, USA.; 15 Department of Clinical Neurobiology, University Hospital Heidelberg and German Cancer Research Center, Heidelberg, Germany.; 16 Department of Neurology, University of California San Francisco, San Francisco, CA, USA.; 17 Department of Cellular and Molecular Medicine, University of California San Diego, La Jolla, CA, USA.; 18 Department of Pediatrics, University of California San Diego, San Diego, CA, USA.; 19 Pathology Service 113B, San Francisco VA Health Care Systems, San Francisco, CA, USA.

## Abstract

Germinal matrix hemorrhage (GMH) is a devastating neurodevelopmental condition affecting preterm infants, but why blood vessels in this brain region are vulnerable to rupture remains unknown. Here we show that microglia in prenatal mouse and human brain interact with nascent vasculature in an age-dependent manner and that ablation of these cells in mice reduces angiogenesis in the ganglionic eminences, which correspond to the human germinal matrix. Consistent with these findings, single-cell transcriptomics and flow cytometry show that distinct subsets of CD45^+^ cells from control preterm infants employ diverse signaling mechanisms to promote vascular network formation. In contrast, CD45^+^ cells from infants with GMH harbor activated neutrophils and monocytes that produce proinflammatory factors, including azurocidin 1, elastase and CXCL16, to disrupt vascular integrity and cause hemorrhage in ganglionic eminences. These results underscore the brain’s innate immune cells in region-specific angiogenesis and how aberrant activation of these immune cells promotes GMH in preterm infants.

Germinal matrix, also known as the ganglionic eminences (GEs), is enriched with neural progenitors that give rise to GABAergic neurons in prenatal human brain^1–3^. Aside from being a neurogenic niche, GEs exhibit active angiogenesis where an ensemble of endothelial and mural cells utilize a repertoire of signaling mechanisms to facilitate cell–cell communication and maturation of the nascent vasculature^4,5^. Interestingly, ~20–40% of preterm infants born before 30 gestational weeks (GW) develop spontaneous hemorrhage in GEs, also known as germinal matrix hemorrhage (GMH), which can cause devastating neurodevelopmental sequelae^6–9^. While immaturity of the brain vasculature and hemodynamic changes have been implicated as possible causes for GMH^10,11^, the exact mechanism remains unclear.

One area of investigation focuses on the brain’s innate immune cells microglia and their contributions to angiogenesis during prenatal brain development. In mice, Runx1^+^ myeloid progenitors colonize the brain parenchyma between embryonic day (E)8.5 and E9.5 and undergo further specification into microglia^12–15^. Fate-mapping and transcriptomic analyses reveal a distinct population of CD206^+^ macrophages that give rise to border-associated macrophages (BAMs) in the mature brain^16,17^. In the prenatal mouse hindbrain, macrophages promote vascular network formation^18^, whereas in postnatal mouse somatosensory cortex (CTX), microglia interact with the established vasculature in areas lacking astrocytic endfeet^19^. Although bacterial infection can activate the microglia inflammasome pathway in neonatal brain^20^, it remains unclear how inflammation disrupts the homeostatic interaction between microglia and blood vessels to promote GMH^10,11^.

Here, we leverage single-cell transcriptomics and high-dimensional cytometry to interrogate the molecular and cellular characteristics of CD45^+^ immune cells isolated from control preterm infants and infants with GMH. Our results show that distinct subsets of CD45^+^ cells employ diverse signaling mechanisms to promote vascular development in GEs during the second trimester. In contrast, CD45^+^ cells from preterm infants with GMH harbor activated neutrophils and monocytes that produce bactericidal factors azurocidin 1 (AZU1), elastase (ELANE) and chemokine CXCL16, which can disrupt vascular integrity and cause hemorrhage in the GEs of embryonic mouse brain. These results reveal previously unappreciated role of the brain’s innate immune cells in region-specific angiogenesis and how aberrant activation of these immune cells lead to GMH in preterm infants.

## Results

### Immune cell–vasculature interactions in the prenatal brain

**Extended Data Fig. 1 |:**
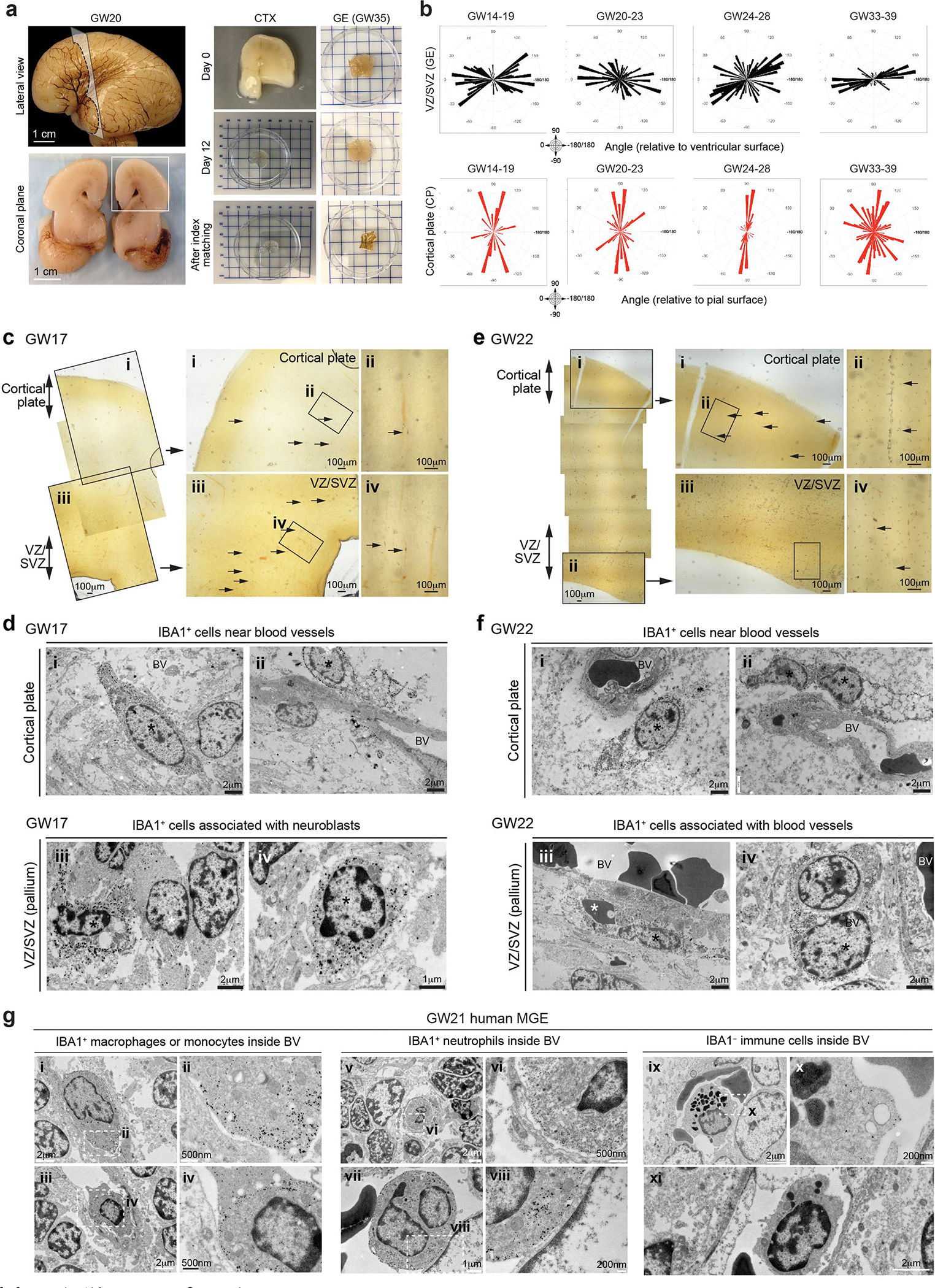
Vector mapping of the nascent vasculature and ultrastructural analyses of their relationship with IBA1^+^ cells in the second trimester human brain. (**a**) Lateral view and coronal plane of prenatal human brain at gestational week 20 (GW20), highlighting the cortex (CTX) section that was used for tissue clearing (left column). A separate block from the ganglionic eminence (GE) was obtained from GW35 prenatal brain (right column). Tissue clearing process and index matching using SHIELD and iDISCO to render thick tissue sections optically transparent (right column). (**b**) Vector mapping of the orientations of the nascent vasculature in the ventricular zone and subventricular zone (VZ/SVZ) in GE and in the cortical plate (CP) at GW14-19, GW20-23, GW24-28, and GW33-39. The results are complied based on 3 independent biological samples for each age group. (**c**) Left panels: Tiled low magnification images of IBA1 immuno-gold stained sections that cover the cortical plate and VZ/SVZ in a GW17 human brain. (Right panels) Higher magnification images of the brain regions in panel c, which highlight the regions of cortical plate (i and ii) and VZ/SVZ (iii and iv) of the pallium. Arrows point to many IBA1^+^ cells near blood vessels or in the brain parenchyma. (**d**) IEM images highlighting IBA1^+^ cells (asterisks) near blood vessels in the cortical plate (i-ii) or their close proximity to neuroblasts in VZ/SVZ in the pallium (iii-iv) at GW17. (**e**) Left panels: Tiled low magnification images of IBA1 immuno-gold stained sections that cover the cortical plate and VZ/SVZ in a GW22 human brain. (Right panels) Higher magnification images of the brain regions in panel e, which highlight the regions of cortical plate (i and ii) and VZ/SVZ (iii and iv) of the pallium. Arrows point to many IBA1^+^ cells near blood vessels or in the brain parenchyma. (**f**) IEM images highlighting IBA1^+^ cells (asterisks) near blood vessels in the cortical plate (i-ii) or blood vessels in VZ/SVZ in the pallium (iii-iv) at GW22. (**g**) IEM images of the medial ganglion eminence (MGE) in a GW21 human brain highlighting IBA1^+^ macrophages or monocytes (panels i-iv), IBA1^+^ neutrophils (v-viii), and IBA1^−^ immune cells (ix-xi) inside blood vessels.

**Fig. 1 |:**
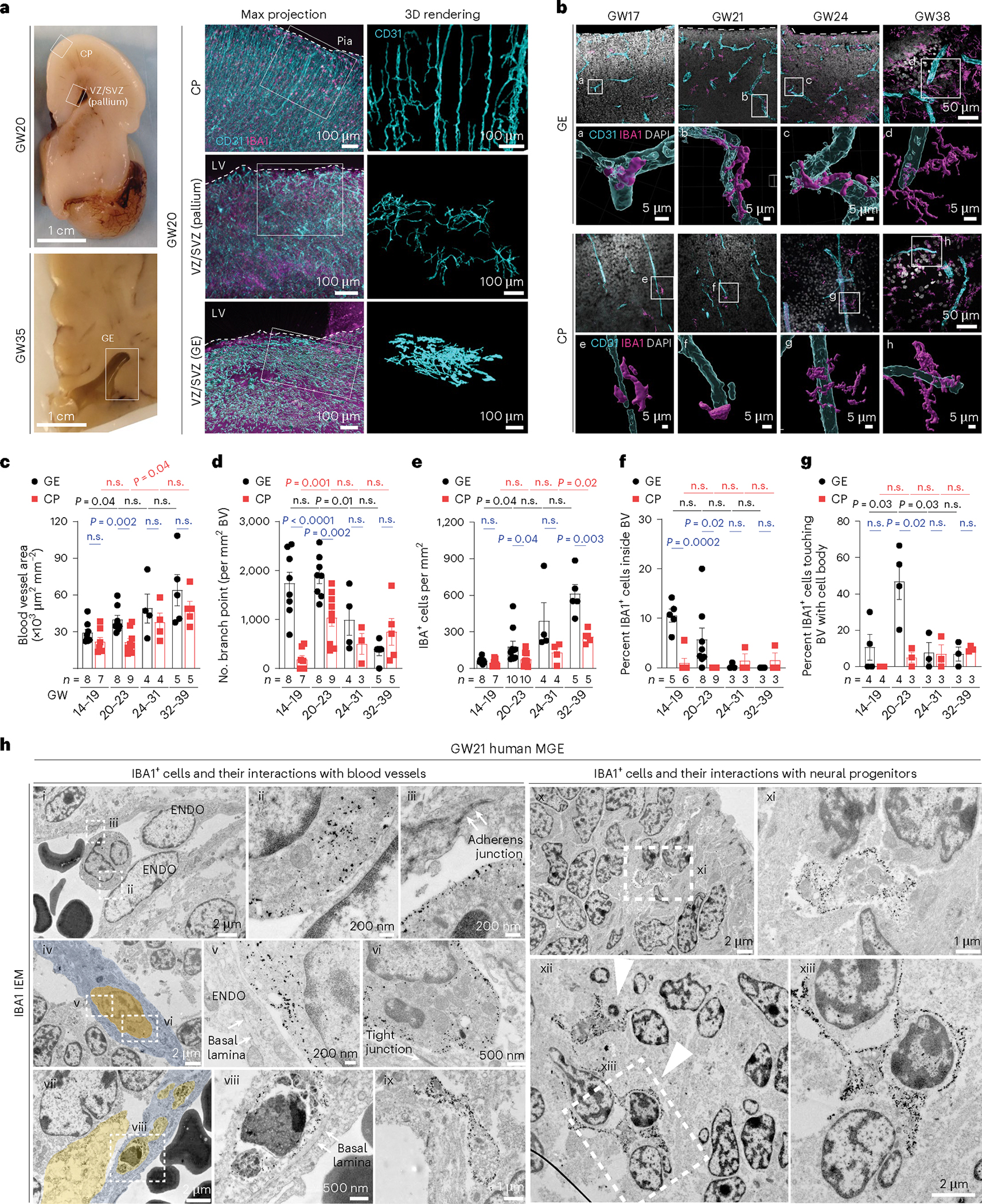
Macrophages/microglia interact with nascent vasculature in the second trimester human brain. **a**, Left: coronal sections of prenatal human brain at GW20 and GW35, highlighting the GEs, VZ/SVZ of the pallium and cortical plate (CP). Middle: the light-sheet images in optically cleared coronal sections show the intricate interactions between IBA1^+^ cells and CD31^+^ endothelial cells in CP, VZ/SVZ of pallium and GEs. Right: IMARIS 3D images reveal the morphology of CD31^+^ endothelial cells in each brain region. LV, lateral ventricle. **b**, Confocal images and IMARIS 3D rendering of IBA1^+^ cells interacting with CD31^+^ endothelial cells in the GE and CP at GW17, GW21, GW24 and GW38. Images highlighted by white boxes are enlarged and represented in IMARIS 3D images in panels below (white letters a–h). **c**,**d**, Quantification of blood vessel and vascular branch point densities in the GE and CP in the prenatal human brain. **e**–**g**, Quantification of the density of IBA1^+^ cells, the percentage of IBA1^+^ cells inside blood vessels and the percentage of extravascular IBA1^+^ cells touching blood vessels with cell body in the GE and CP. **h**, IEM using the IBA1 antibody shows macrophages and microglia inside blood vessels with primitive basal lamina and in the perivascular milieu in the MGE of prenatal human brain at GW21. The arrows in (iii) indicate primitive adherens junction in endothelial cells, and the arrowheads in (xii) indicate IBA1^+^microglia engulfing neuroblasts. The images in **h** are from one GW21 prenatal human brain. ENDO, endothelial cell. The same experiments were performed in another second trimester case at GW17 with the similar results. Statistics in **c**–**g** use a two-tailed, unpaired Student’s *t*-test, and the data represent the mean ± standard error of the mean. n.s., not significant. *n* indicates the number of independent biological samples used for quantification.

To characterize the interaction between microglia and blood vessels in the prenatal human brain, we used light-sheet microscopic imaging on optically cleared coronal sections from the germinal matrix and frontal CTX at GW20 and GW35 (Extended Data Fig. 1a and Supplementary Table 1). Using IBA1 as a generic marker for macrophages/microglia and CD31 for endothelial cells, we showed that blood vessels in the cortical plate were oriented perpendicular to the pial surface, whereas blood vessels in the ventricular zone and subventricular zone (VZ/SVZ) of the pallium and GEs exhibited complex branches parallel to the ventricular surfaces (Fig. 1a,b and Extended Data Fig. 1b). Notably, the blood vessel area showed age-dependent increases in both regions but was significantly higher in the GEs compared with the cortical plate at GW20–23 (Fig. 1c). In addition, the vascular branch point density in the GEs was higher than those in the cortical plate at GW14–23 (Fig. 1d). Correlatively, IBA1^+^ cell density was much higher in GEs at GW20–23 (Fig. 1e). A higher percentage of IBA1^+^ cells was identified inside the blood vessels in the VZ/SVZ of GEs than those in the cortical plate at GW14–23, whereas more extravascular IBA1^+^ cells directly touched the blood vessels in the VZ/SVZ of GEs at GW20–23 (Fig. 1b,f,g). Consistent with these results, immunogold electron microscopy (IEM) using IBA1 antibody captured different features of IBA1^+^ cells in relation with blood vessels, including those traveling near, through or embedded within endothelial cells in cortical plate, VZ/SVZ of the pallium and GEs at GW17–22 (Fig. 1h(i–viii) and Extended Data Fig. 1c–g), and those resided in the perivascular spaces in GE (Fig. 1h(ix)). Within GE, IBA1^+^ microglia were adjacent to or surrounding neural progenitors or neuroblasts (Fig. 1h(x–xiii)). Together, these results suggested a continuous transition of IBA1^+^ cells from inside the vascular lumen to perivascular spaces that was more prominent in the VZ/SVZ of GEs during prenatal human brain development.

### Macrophages/microglia promote angiogenesis in mouse GE

**Extended Data Fig. 2 |:**
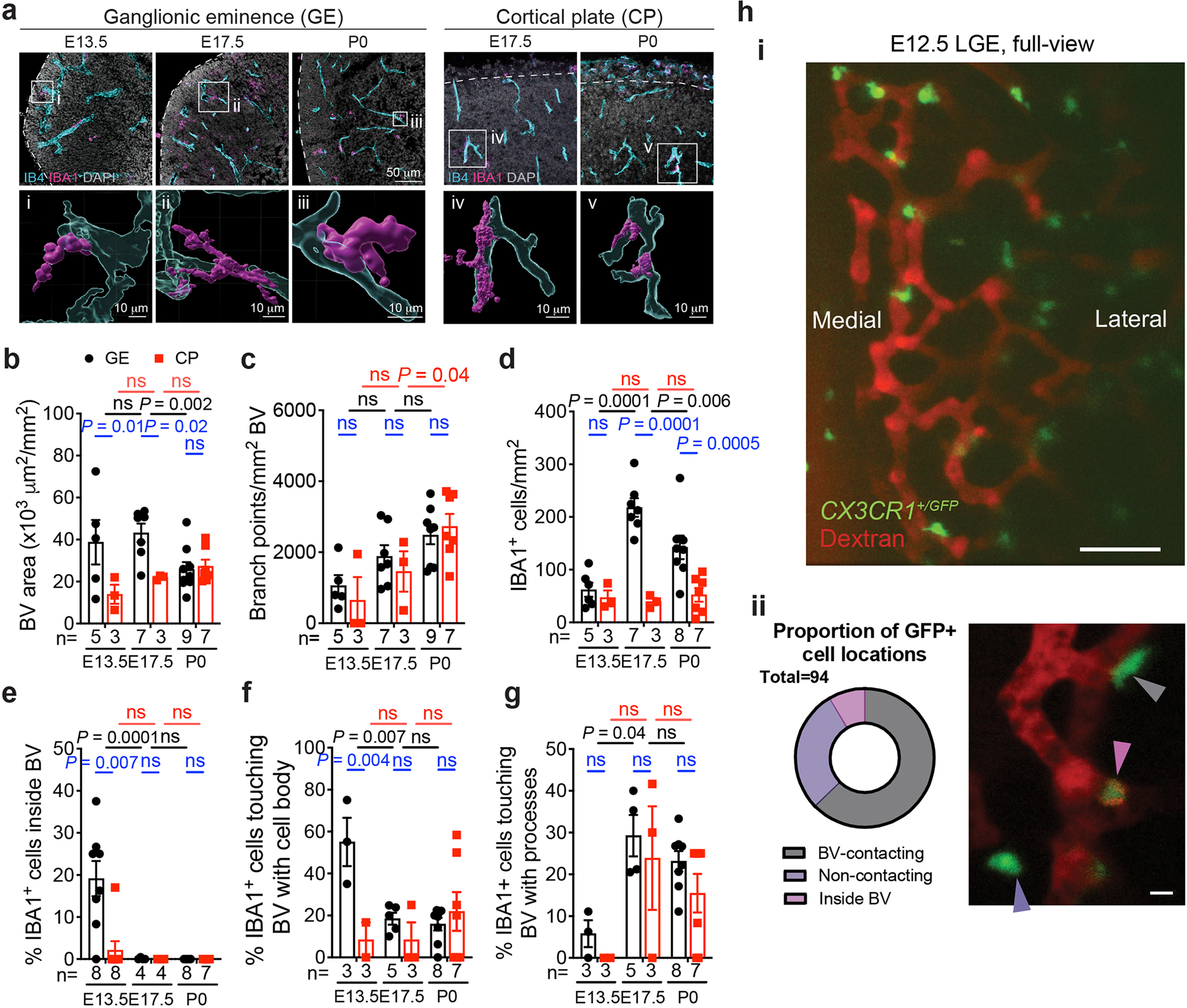
Macrophages/ microglia cells interact with nascent vasculature in the embryonic mouse brain. (**a**) Confocal images and IMARIS 3D rendering of IBA1^+^ cells interacting with IB4^+^ vasculature in GE and CP at E13.5, E17.5, and P0 in pre- and perinatal mouse brains. (**b, c**) Quantification of blood vessel and vascular branch point densities in GE and CP in the mouse brain. (**d–g**) Quantification of the density of IBA1^+^ cells, the percentage of IBA1^+^ cells inside blood vessels, and the percentage of IBA1^+^ cells touching the external surface of blood vessels with cell body or processes in GE and CP in the mouse brain. The number below each bar represents the number of biological replicates analyzed in each experiment. (**h**) Still image highlighting the full view of the lateral ganglionic eminence (LGE) in an E12.5 mouse embryo undergoing *ex utero* live imaging (i). A representative image showing three different behaviors of *Cx3cr1*^+/*GFP*^ cells and quantification of these three different cell behaviors from a total of 97 cells from 9 mouse embryoes captured in live imaging. Statistics in panels **b-g** use two-tailed, unpaired Student’s t-test, data represent mean ± SEM. ns, not significant. *n* indicates the number of independent biological samples used for quantification.

**Fig. 2 |:**
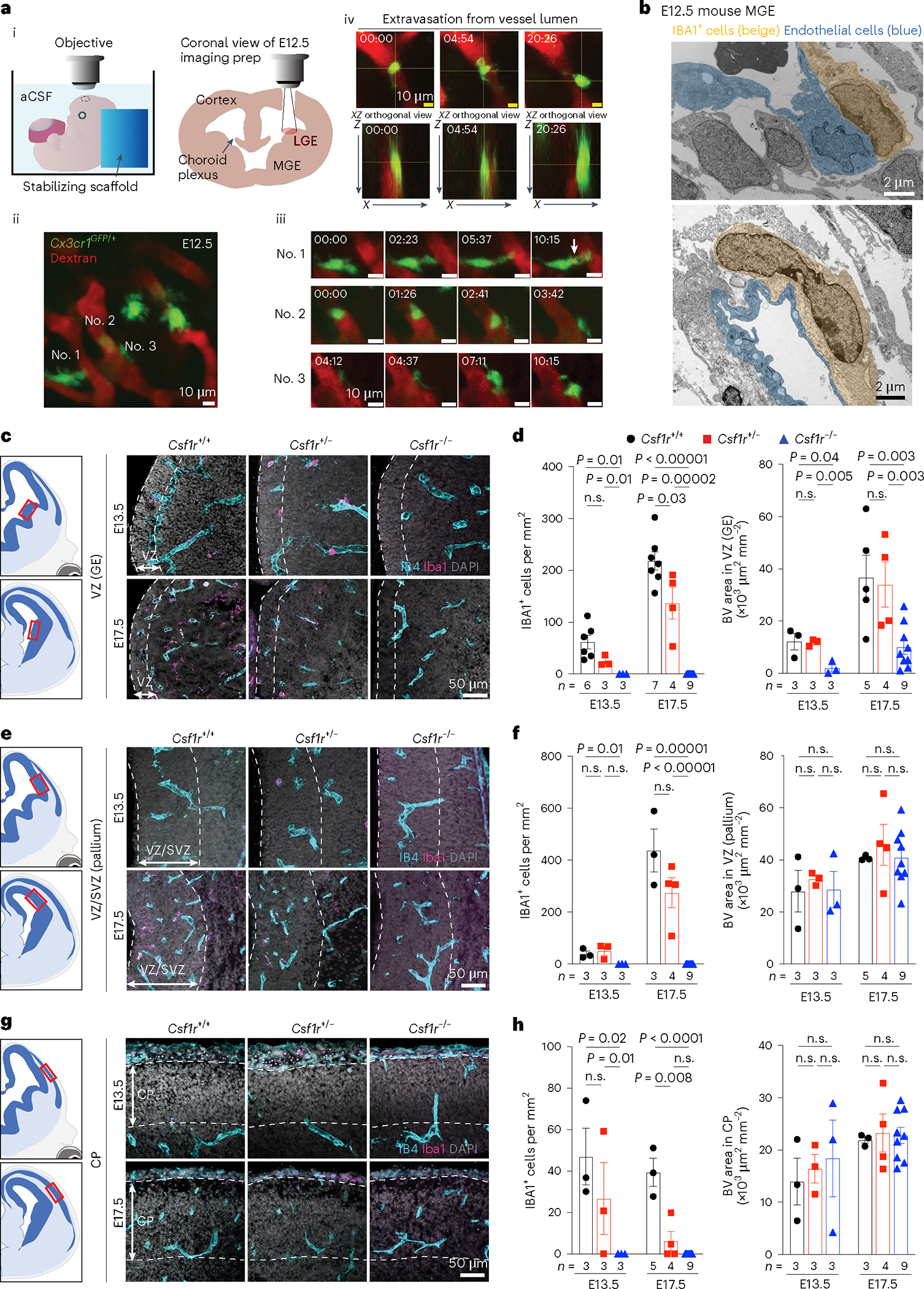
Macrophages/microglia are required for angiogenesis in the ventricular zone of the GEs. **a**. Live imaging of *Cx3cr1*^+/*GFP*^ macrophages/microglia with nascent vasculature in the GEs. (i) Schematic diagrams of imaging setup for E12.5 LGE in *Cx3cr1*^+/*GFP*^ mice. The embryo remains attached to placenta while bathed in artificial cerebrospinal fluid (aCSF). (ii) Max projection of an in vivo two-photon image with *Cx3cr1*^+/*GFP*^ cells in LGE associated with blood vessels illuminated with Texas Red dextran. (iii) Still frames of timelapse images of three highlighted *Cx3cr1*^+/*GFP*^ cells, with no. 1 extending the process into the blood vessel and taking up dextran (white arrow), no. 2 rolling within the blood vessel before releasing into the circulation and no. 3 moving along the surface of a blood vessel. (iv) Extravasation of a *Cx3cr1*^+/*GFP*^ cell between the lumen and abluminal side. Orthogonal views show the macrophage against one vessel wall on the luminal side at time 0 min and against the vessel wall on the abluminal side at 20:26 min. **b**, IEM using IBA1 antibody shows IBA1^+^ macrophages and microglia directly attached to the endothelial cells in MGE of E12.5 mouse brain. This was performed in three biological replicates. **c**,**e**,**g**, Loss of CSF1R leads to complete ablation of IBA1^+^ cells in GE (**c**), VZ/SVZ of the pallium (**e**) and the cortical plate (CP) (**g**). The red boxes in the schematic diagrams show the regions captured in confocal images. **d**,**f**,**h**, Quantification of densities of IBA^+^ cells and IB4^+^ blood vessels in the VZ of GE (**d**), the SVZ/VZ of pallium (**f**) and the CP (**h**). The dashed lines indicate the regions in which blood vessel quantifications are performed. Statistics in **d**, **f** and **h** use a two-tailed, unpaired Student’s *t*-test, and the data represent the mean ± standard error of the mean. n.s., not significant. *n* indicates the number of independent biological samples used for quantification.

Similar to human GEs at GW20–23, the blood vessel density in the E13.5–17.5 mouse brain was higher in the GEs than in the cortical plate (Extended Data Fig. 2a,b). Although vascular branch point density did not differ between the two regions, the density of IBA1^+^ cells was higher in GEs than in the cortical plate at E17.5 and postnatal day 0 (P0) (Extended Data Fig. 2c,d). Furthermore, a higher percentage of IBA1^+^ cells resided inside the vascular lumen or touching blood vessels in GEs at E13.5 than in the cortical plate (Extended Data Fig. 2e–g). To determine whether microglia precursors can undergo transendothelial migration from the circulation into the brain parenchyma, we conducted ex vivo live imaging in the lateral GE (LGE) of E12.5 *Cx3cr1*^+/*GFP*^ reporter mice while visualizing blood vessels via intraplacental injection of Texas Red dextran (Fig. 2a(i))^21^. Consistent with its role in immune surveillance, many *Cx3cr1*^+/*GFP*^ cells were highly motile near the perivascular region in the LGE (Fig. 2a(ii–iii) and Supplementary Video 1), with some extending processes into the vascular lumen to engulf dextran (Fig. 2a(iii)). A total of 8 of 94 *Cx3cr1*^+/*GFP*^ cells (recorded across nine embryos) were found in the vessel proper, and at least one crossed the primitive blood–brain barrier (BBB) in LGE at E12.5 (Fig. 2a(iv) and Supplementary Video 2). Over half of the recorded *Cx3cr1*^+/*GFP*^ cells in LGE contacted blood vessels (Extended Data Fig. 2h and Supplementary Video 3). Consistent with these results, IEM showed that IBA1^+^ cells directly contacted endothelial cells with primitive adherens junction and lacked definitive basement membrane in medial GE (MGE) at E12.5 (Fig. 2b).

**Extended Data Fig. 3 |:**
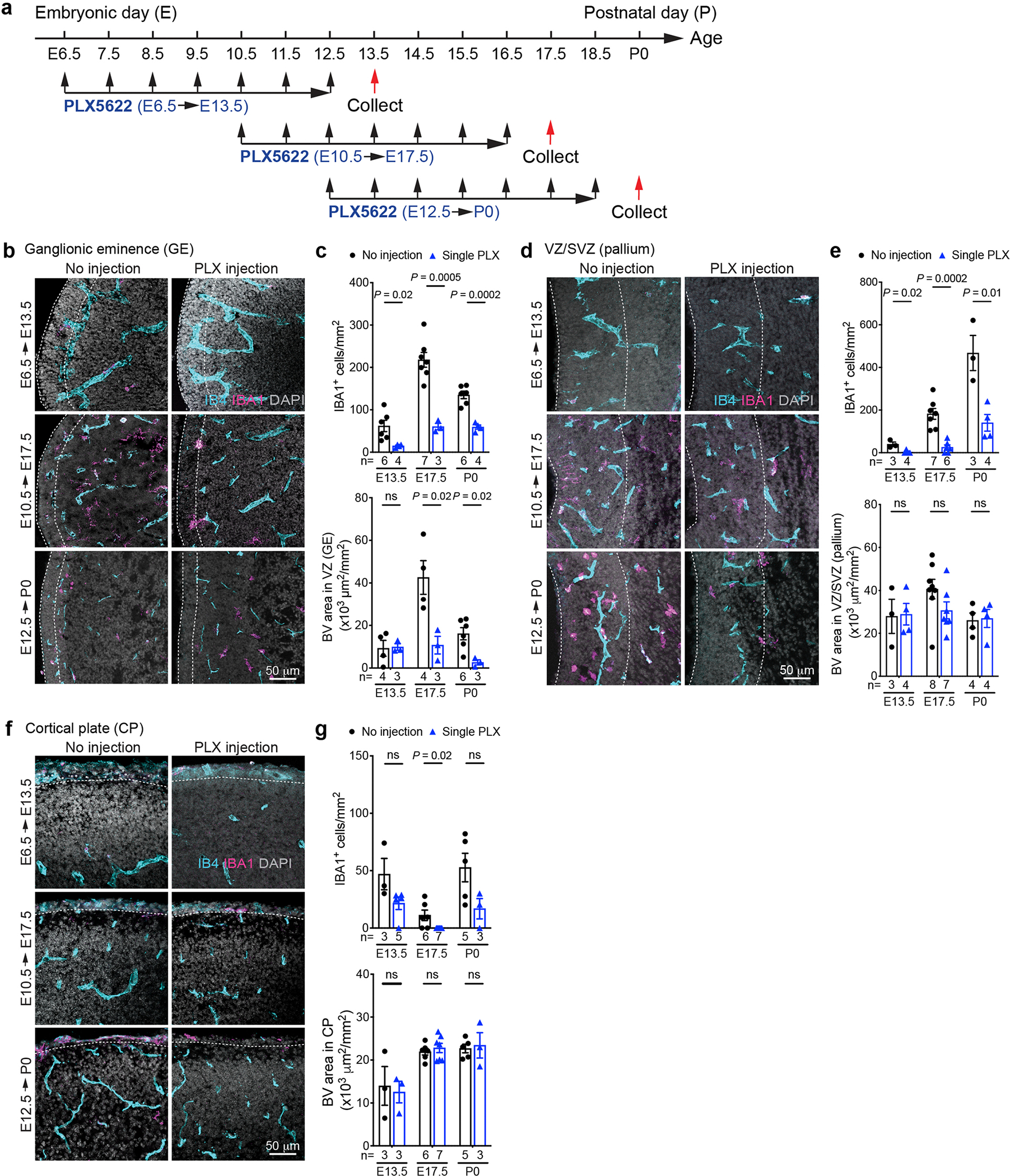
Macrophage/ microglia promote angiogenesis in the ventricular zone (VZ) of the ganglionic eminences. (**a**) Schematic diagram showing three schedules of CSF1R inhibitor PLX5622 injection in pregnant mouse dams. (**b**, **d**, **f**) Transient inhibition of CSF1R leads to significant depletion of IBA1^+^ cells in the VZ of GE (**b**), VZ/SVZ of the pallium (**d**), and the CP (**f**). (**c**, **e**, **g**) Quantification of densities of IBA^+^ cells and IB4^+^ blood vessels in the VZ of GE (**c**), VZ/SVZ of the pallium (**e**), and the CP (**g**). Dashed lines indicate the regions in which quantifications are performed. Statistics in panels **c**, **e**, and **g** use two-tailed, unpaired Student’s t-test, data represent mean ± SEM. ns, not significant. *n* indicates the number of independent biological samples used for quantification.

To determine how macrophages/microglia affect angiogenesis, we deleted the mouse *Csf1r* gene, which supports the survival of these cells^22^. As expected, *Csf1r*^*−/−*^ embryos showed a complete loss of IBA1^+^ cells in GE, pallium and cortical plate at E13.5 and E17.5, while IBA1^+^ cells were partially depleted in *Csf1r*^+/*−*^ embryos. Intriguingly, *Csf1r*^−/−^ embryos showed reduced vascular density in the VZ of GE but not in VZ/SVZ of pallium or in the cortical plate (Fig. 2c–h). To determine whether microglia have stage-dependent effects on angiogenesis, we intraperitoneally injected wild-type pregnant dams with CSF1R inhibitor PLX5622 (50 mg kg^−1^) for seven consecutive days starting at E6.5, E10.5 or E12.5 to deplete myeloid cells. We then collected embryos 1 day after the last injection at E13.5, E17.5 or P0 (Extended Data Fig. 3a). All three regimens significantly reduced IBA1^+^ cell density in the GE, pallium and cortical plate (Extended Data Fig. 3b–g). Interestingly, IBA1^+^ cell depletion from E6.5 to E12.5 did not alter vascular density in the VZ of GE, whereas depletion from E10.5 to E16.5 or from E12.5 to E18.5 significantly reduced vascular density in the same region (Extended Data Fig. 3c). In contrast, IBA1^+^ cell depletion did not decrease vascular density in the VZ/SVZ of pallium or cortical plate (Extended Data Fig. 3d–g). These results support that microglia and macrophages have region-specific and age-dependent effects in promoting angiogenesis in GEs.

### CD45^+^ cells and angiogenesis in prenatal human brain

**Extended Data Fig. 4 |:**
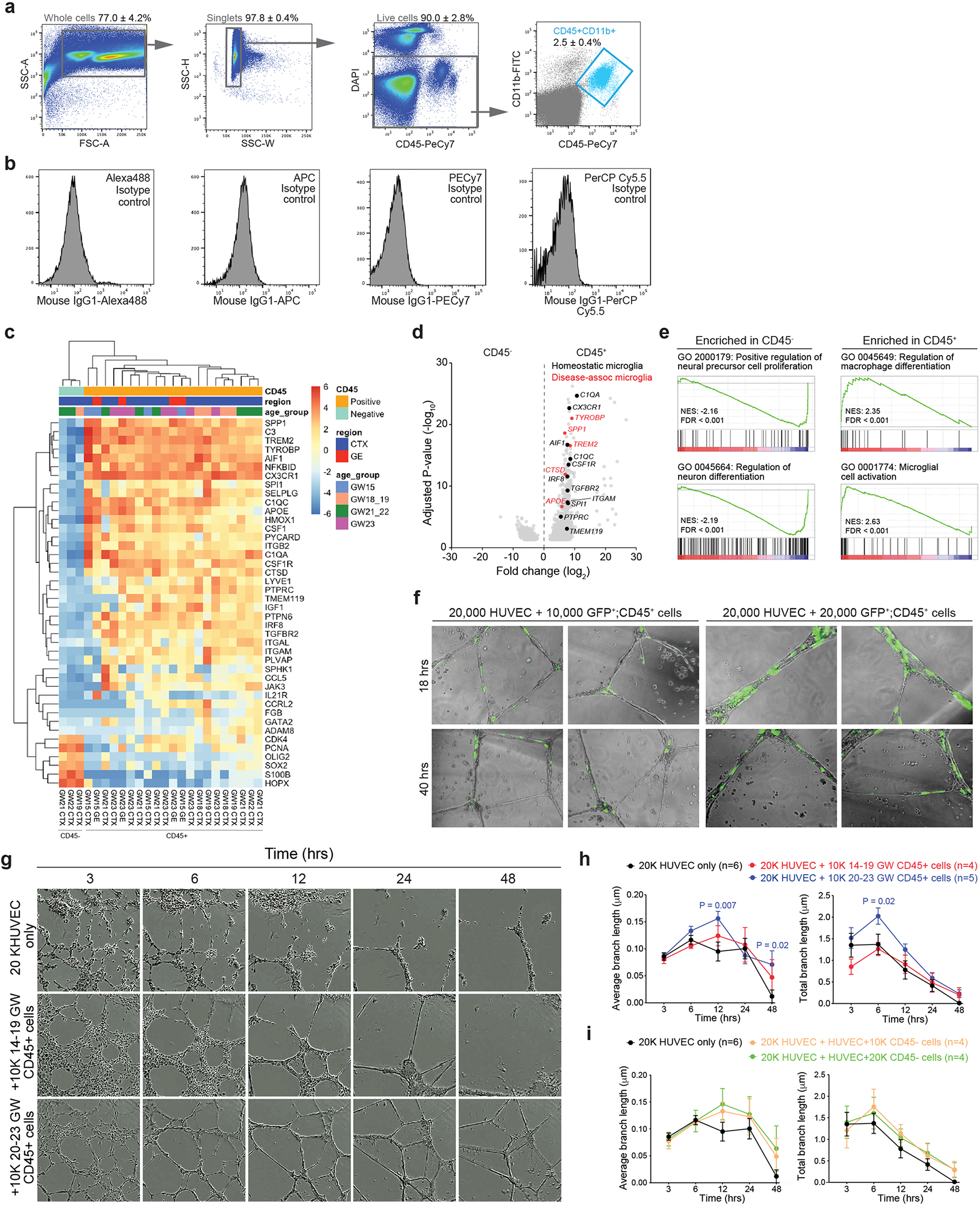
Stage-dependent role of human CD45^+^ immune cells in promoting vascular morphogenesis. (**a**) Fluorescence-activated cell sorting (FACS) gating strategy to select for CD45^+^;CD11b^+^ cells. (**b**) Isotype controls used in FACS. (**c**) Heatmap of critical differentially expressed genes in CD45^−^ and CD45^+^ cells used in bulk RNA-seq. (**d**) Volcano plot shows the genes enriched in CD45^+^ cells (right). Genes shown were filtered to be below adjusted p-value of 0.05 and above fold change of 1.2 between CD45^−^ and CD45^+^ cells. (**e**) Gene set enrichment analysis (GSEA) reveals gene ontology (GO) terms enriched in CD45^−^ and CD45^+^ cells. Data from panels **c-e** are from 3 independent biological samples of CD45^−^ cells, and 21 independent biological samples of CD45^+^ cells. (**f**) Images taken from InCucyte S3 Live Imaging Device of HUVEC and AAV-CMV-GFP transfected CD45^+^ cells from prenatal human brain samples at 18 and 40 hrs after plating. (**g**) Images taken from InCucyte S3 Live Imaging Device of HUVEC at 3, 6, 12, 24 and 48 hrs after plating. The conditions include 20,000 HUVEC alone, and 20,000 HUVEC co-cultured with 10,000 GW14-19 or GW20-23 CD45^+^ cells from prenatal human brain. (**h, i**) Quantification of average and total endothelial branch lengths formed by HUVEC that are co-cultured with CD45^+^ cells (**h**) or CD45^−^ cells (**i**). Statistics in panels h and i use two-tailed, unpaired Student’s t-test, data represent mean ± SEM. The *P* values represent comparisons between HUVECs co-incubated with CD45^+^ cells *vs* HUVECs only. Not significant comparisons are not shown. *n* indicates the number of independent biological samples used for quantification.

**Fig. 3 |:**
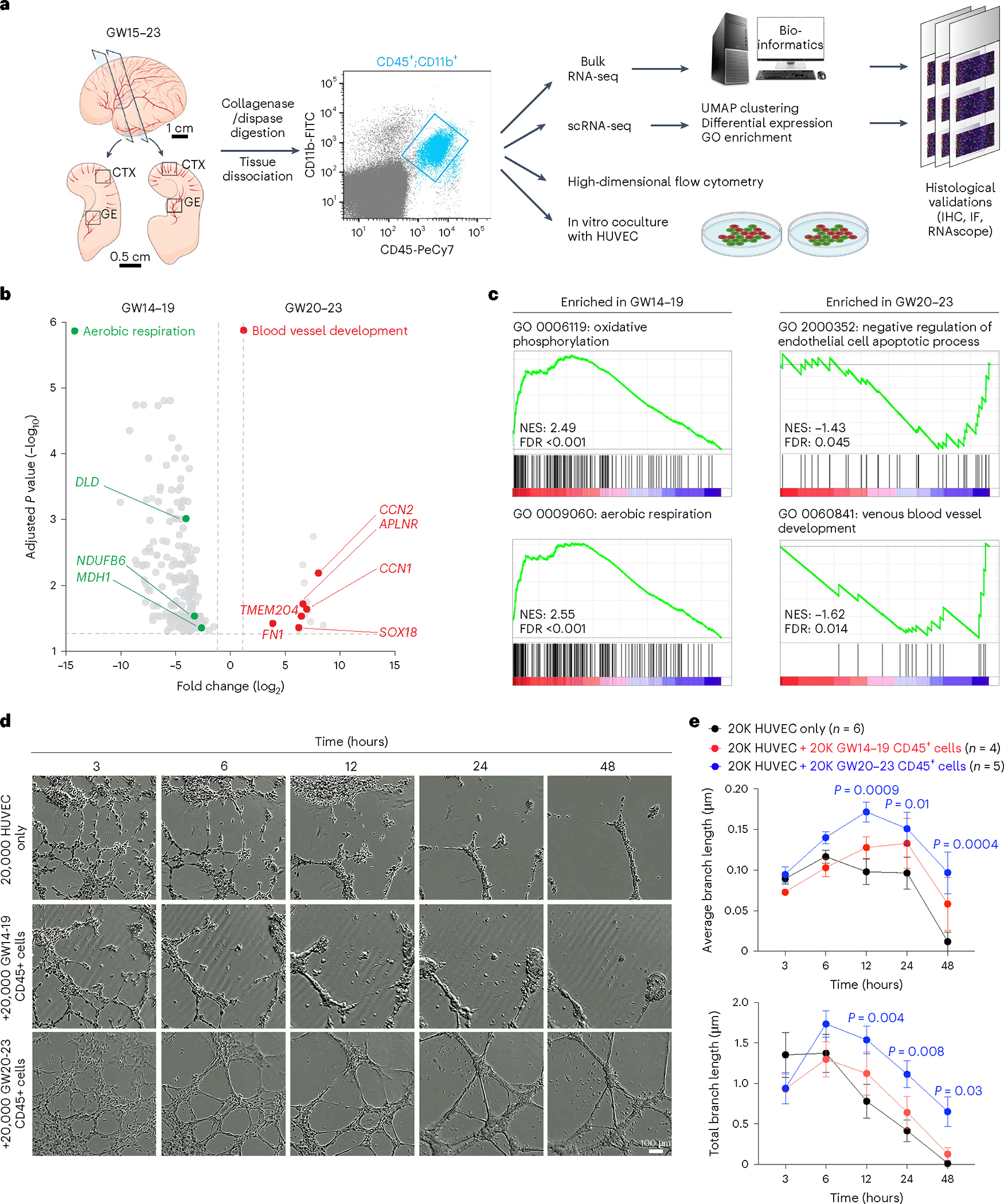
Stage-dependent role of CD45^+^ immune cells in promoting vascular morphogenesis. **a**, Schematic diagrams showing the strategy to isolate CD45^+^;CD11b^+^ immune cells from the CTX and GE of prenatal human brain from GW15–23. These CD45^+^;CD11b^+^ cells are subjected to bulk RNA-seq and scRNA-seq, followed by bioinformatics analyses. The transcriptomic data are validated using immunohistochemistry (IHC), immunofluorescence microscopy (IF) and RNAscope-based in situ hybridization. Finally, CD45^+^;CD11b^+^ cells are further characterized using high-dimensional flow cytometry and 3D Matrigel HUVEC assays. **b**, A volcano plot showing the genes enriched in CD45^+^ cells from GW20–23 (right) and those enriched in cells from GW14–19 (left). Adjusted *P* values and fold changes were calculated using DESeq2. By default in DESeq2, the *P* values attained by the Wald test are corrected for multiple testing using the Benjamini–Hochberg method. The genes shown were filtered to be below the adjusted *P* value of 0.05 and above a fold change of 1.2 between GW14–19 and GW20–23 comparisons (highlighted by the dashed lines). **c**, GSEA reveals GO terms enriched in CD45^+^ cells from GW14–19 and GW20–23. The data in **b** and **c** are from 21 independent biological samples. NES, normalized enrichment score; FDR, false discovery rate. **d**, Images taken from InCucyte S3 Live Imaging Device of HUVEC in Matrigel-based branching morphogenesis at 3, 6, 12, 24 and 48 h after plating. The conditions include 20,000 HUVEC alone and 20,000 HUVEC cocultured with 20,000 GW14–19 or GW20–23 CD45^+^ cells from prenatal human brain. **e**, Quantification of average and total endothelial branch lengths formed by HUVEC. Statistics use a two-tailed, unpaired Student’s *t*-test, and the data represent the mean ± standard error of the mean. The *P* values represent comparisons between HUVEC coincubated with CD45^+^ cells versus HUVEC only. Not significant comparisons are not shown. *n* indicates the number of independent biological samples used for quantification. For each biological sample, at least three technical replicates are used.

To determine whether microglia and their progenitors could also regulate vascular development in prenatal human brain, we used fluorescence-activated cell sorting (FACS) to isolate CD45^+^ cells from the GE and cortical plate at GW15–23 and subjected these cells for bulk and single-cell RNA sequencing (scRNA-seq), high-dimensional flow cytometry, and coculture with human umbilical vein endothelial cells (HUVEC) in Matrigel (Fig. 3a). For bulk RNA sequencing (RNA-seq), we collected CD45^+^ cells from 17 samples from the CTX and four samples from the GE and CD45^−^ cells from three samples (as negative controls) (Extended Data Fig. 4a,b and Supplementary Table 1b). Our results showed that CD45^+^ cells were highly enriched in canonical microglia genes, including *AIF1, TMEM119*, *SPI1*, *CX3CR1*, *CSF1R* and *IRF8* (Extended Data Fig. 4c,d) and genes identified in other subtypes of microglia, including *LYVE1* (BAM), *TREM2* and *APOE* (disease-associated microglia)^17,23,24^. In contrast, CD45^−^ cells were enriched in genes related to proliferation (*CDK4* and *PCNA*) or neural progenitors (*OLIG2*, *SOX2* and *HOPX*) (Extended Data Fig. 4c and Supplementary Table 2). Gene set enrichment analysis (GSEA) confirmed that CD45^−^ cells consisted of radial glia, immature neurons and oligodendrocytes, whereas CD45^+^ cells mostly consisted of microglia and macrophages that expressed genes related to cell adhesion (*ITGB2*, *ITGAL*, *ITGAM* and *ADAM8*), chemotaxis (*CCRL2* and *CCL5*) and endothelial cell-related functions (*PLVAP* and *GATA2*)^4^ (Extended Data Fig. 4e). In addition, GW14–19 CD45^+^ cells showed higher expression of genes related to aerobic respiration (*DLD*, *NDUFB6* and *MDH1*), whereas GW20–23 CD45^+^ cells showed enrichment of genes involved in blood vessel development (*SOX18*, *CCN1*, *CCN2*, *APLNR*, *FN1* and *TMEM204*) (Fig. 3b,c and Supplementary Table 3).

To test whether CD45^+^ cells could promote vascular development, we cocultured CD45^+^ cells with HUVEC in Matrigel. To visualize CD45^+^ cells, we incubated them with AAV–CMV–GFP (Extended Data Fig. 4f). In the absence of CD45^+^ cells, HUVEC showed modest branching morphogenesis at 20,000 cells per well (Supplementary Video 4). Interestingly, adding 10,000 or 20,000 CD45^+^ cells from GW20–23 prenatal human brain to 20,000 HUVEC increased both average and total branch length even after 48 h in culture, suggesting a role for CD45^+^ cells in promoting vascular branching morphogenesis (Fig. 3d,e, Extended Data Fig. 4g,h and Supplementary Video 5). In contrast, CD45^+^ cells or CD45^−^ cells from GW14–19 human brain samples did not enhance branching morphogenesis in HUVEC (Fig. 3d,e, Extended Data Fig. 4g–i and Supplementary Videos 6 and 7).

### scRNA-seq reveals GE-enriched CD45^+^ cell subtypes

**Extended Data Fig. 5 |:**
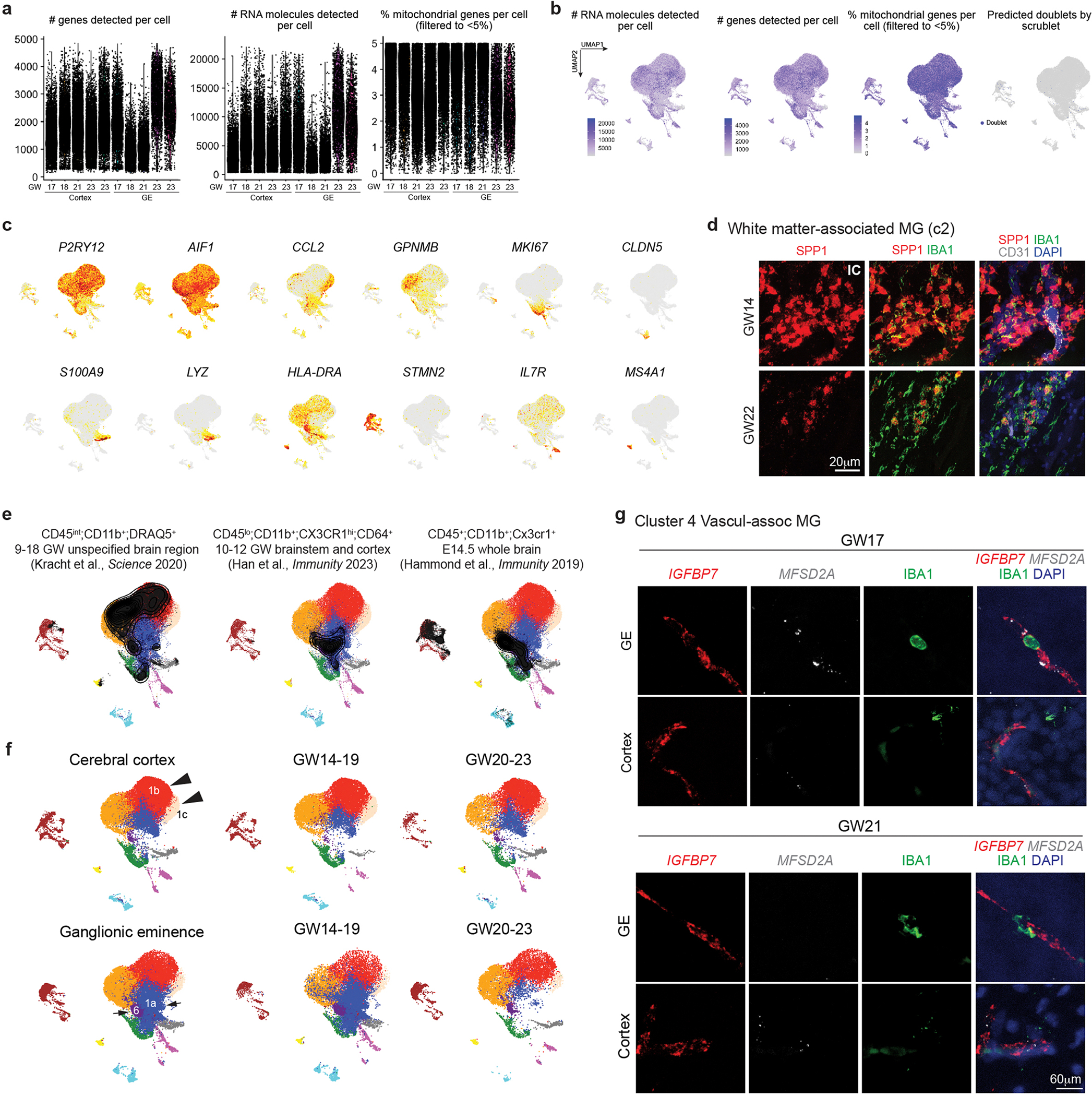
Single-cell transcriptomics reveal subtypes of CD45^+^ cells and their regional specificity. (**a-b**) Quantifications of the number of genes, RNA molecules, and percentage of mitochondrial genes per cell, as well as predicted doublets from scRNA-seq. Each dot represents one cell. (**c**) UMAP feature plots of marker gene expressions that define each subtype of CD45^+^ cells. (**d**) Confocal images of white matter-associated microglia marker *SPP1* (RNAscope probe) in IBA1^+^ cells in the internal capsule (IC) of GW14 and GW22 human brains. (**e**) Projection of published scRNA-seq datasets of microglia in prenatal human and mouse brain onto the UMAP plot of our scRNA-seq. (**f**) UMAP plots show differences in the clustering of CD45^+^ cell subtypes from the cerebral cortex at GW14-19 and GW20-23 (arrowheads), and the clustering of CD45^+^ cell subtypes from the ganglionic eminences at GW14-19 and GW20-23 (arrows). Data from panels **a-c**, **e**, and **f** are from 5 independent biological samples. (**g**) Confocal images of VAM markers *IGFBP7* and *MFSD2A* (RNAscope probes) in IBA1^+^ cells in GE and cortex of GW17 and GW21 human brains. The experiments in panels **d** and **g** were repeated in three independent biological replicates.

**Fig. 4 |:**
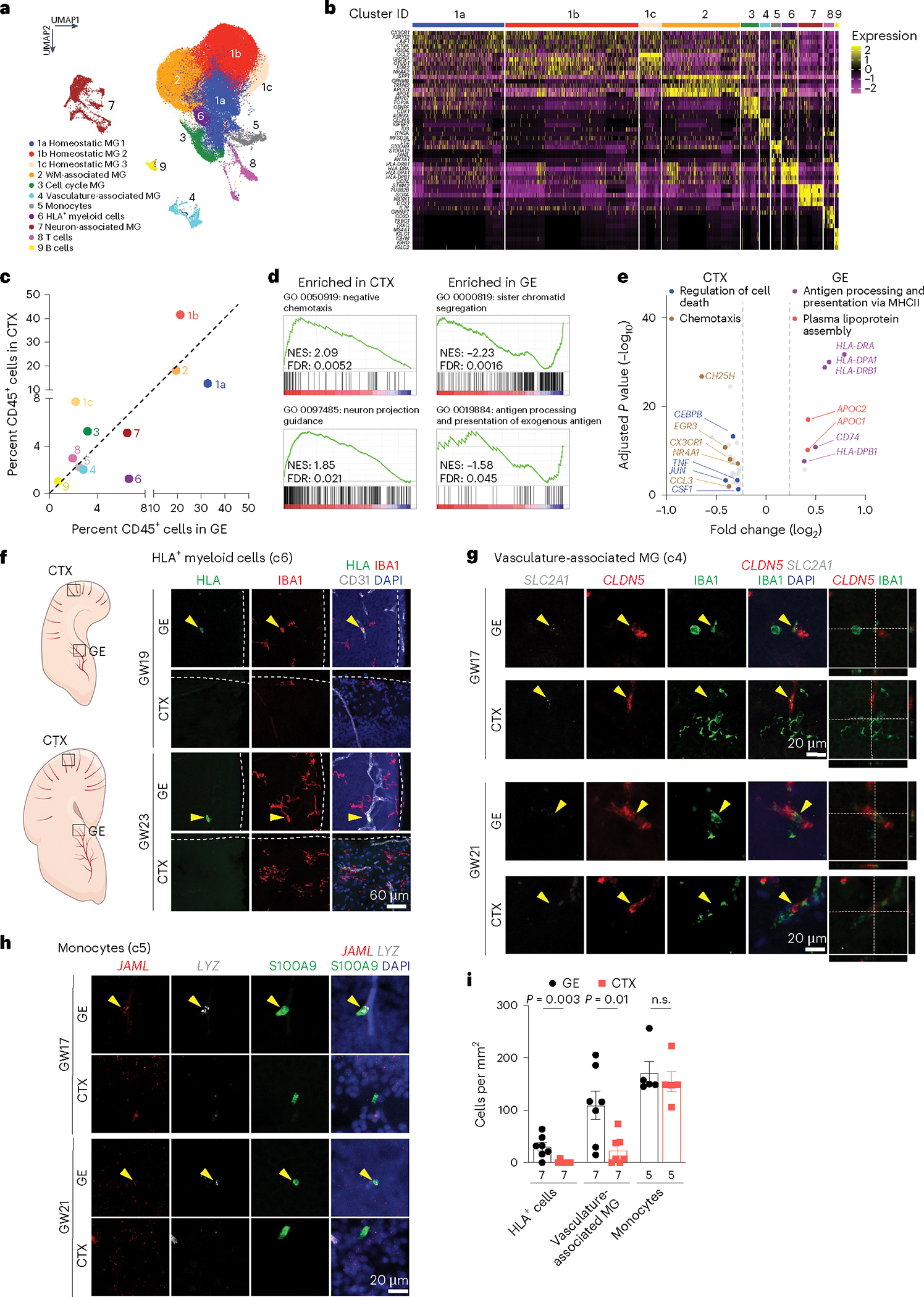
Single-cell transcriptomics reveal subtypes of CD45^+^ cells and their interactions with endothelial cells in prenatal human brain. **a**, UMAP plot highlighting 11 distinct CD45^+^ cell subtypes. **b**, A heat map of marker gene expressions that define each subtype of CD45^+^ cells. MG, microglia; WM, white matter. **c**, A distribution plot comparing the relative abundance of each CD45^+^ subtype in GE versus CTX. **d**, GSEA analysis of the bulk RNA-seq data reveal GO terms defined by genes enriched in GE versus CTX. **e**, A volcano plot showing DEGs identified by pseudobulked scRNA-seq data in GE versus CTX and the GO terms they define. Adjusted *P* values and fold changes were calculated using DESeq2. By default in DESeq2, the *P* values attained by the Wald test are corrected for multiple testing using the Benjamini–Hochberg method. The genes shown were filtered to be below the adjusted *P* value of 0.05 and above a fold change of 1.2 between CTX and GE comparisons (highlighted by the dashed lines). The data in **a**–**c** and **e** are from five independent biological samples at GW17–23. The data in **d** are from 21 independent biological samples at GW15–23. **f**, Confocal images from GE and CTX of GW19 and GW23 human brain validating the presence of HLA^+^ cells in GE (yellow arrowheads) but not in CTX. White lines in ‘GE’ panels indicate the ventricular surface, whereas white lines in ‘Cortex’ panels indicate the pia surface. **g**, Confocal images of VAM markers *SCL2A1* and *CLDN5* (RNAscope probes) in IBA1^+^ cells in GE (yellow arrowheads) but not in CTX of GW17 and GW21 human brains. White lines indicate the section planes for the orthogonal views of CLND5^+^; IBA1^+^ vasculature-associated MG (right and bottom panels). **h**, Confocal images of monocyte markers *JAML* and *LYZ* (RNAscope probes) in S100A9^+^ cells in GE (yellow arrowheads) but not in CTX of GW17 and GW21 human brains. **i**, Quantification of the density of HLA^+^ cells, VAM and monocytes in GE and CTX of prenatal human brain. n.s., not significant. Statistics in **i** use a two-tailed, unpaired Student’s *t*-test, and the data represent the mean ± standard error of the mean. *n* indicates the number of independent biological samples used for quantification.

To further characterize CD45^+^ subtypes, we performed scRNA-seq on FACS-isolated CD45^+^ cells from GE and cortical plate at GW17–23 (Supplementary Table 1c). After quality control, we obtained 60,595 cells, with 56,108 reads and 1,654 genes per cell (Extended Data Fig. 5a,b). Clustering identified 11 subtypes, including 3 homeostatic microglia subtypes (c1a, c1b and c1c), white-matter-associated microglia (c2), cell cycle microglia (c3), vasculature-associated microglia (VAM, c4), monocytes (c5), HLA^+^ myeloid cells (c6), neuron-associated microglia (c7), T cells (c8) and B cells (c9) (Fig. 4a,b and Supplementary Table 4). The homeostatic microglia clusters were enriched in canonical microglia genes such as *AIF1* and *CX3CR1* (Fig. 4b and Extended Data Fig. 5c). White-matter-associated microglia (c2) were transcriptomically similar to the previously defined proliferative region-associated microglia (*SPP1*, *GPNMB* and *CLEC7A*)^23,25^ and disease-associated microglia (*TREM2*)^24^ and were abundant near white matter tracts in the internal capsule (Extended Data Fig. 5d). Cell cycle microglia (c3) were enriched in proliferation genes (*MKI67*, *TOP2A* and *AURKA*), whereas T and B cells showed high-level expressions of *IL7R* and *MS4A1*, respectively. Neuron-associated microglia (c7) were enriched in neuronal genes (*STMN2*, *NRXN1*, *DCX* and *CAMK2N1*), as previously reported^23,25^ (Fig. 4b and Extended Data Fig. 5c). VAM (c4) expressed canonical microglia genes, such as *P2RY12* and *AIF1*, and endothelial genes (*CLDN5*, *MFSD2A*, *SLC2A1*, *ITM2A*, *IGFBP7)*, which could potentially enable these microglia to migrate along the vasculature or across the primitive BBB (Fig. 1h(i–ix) and Extended Data Fig. 5c). Finally, monocytes (c5) expressed *LYZ*, *JAML* and *S100A9*, and HLA^+^ myeloid cells (c6) expressed *CD74*, *HLA-DP* and *HLA-DR* (Fig. 4b and Extended Data Fig. 5c), though *S100A9* has been detected in neutrophils in other single-cell datasets^26,27^.

Next, we projected published datasets on embryonic microglia, including CD45^int^;CD11b^+^;DRAQ5^+^ cells from GW9–18 human brain^28^, CD45^lo^;CD11b^+^;CX3CR1^hi^;CD64^+^ cells from GW10–12 brainstem and CTX^29^ and CD45^+^;CD11b^+^;Cx3cr1^+^ cells from E14.5 mouse brain^23,25^, onto our scRNA-seq Uniform Manifold Approximation and Projection (UMAP) (Extended Data Fig. 5e). These comparisons confirmed the broad repertoire of immune cells captured in our dataset. We also compared the relative abundance of immune cell subtypes in the GE and CTX and found that c1a homeostatic microglia and c6 HLA^+^ myeloid cells were preferentially located in the GE, whereas homeostatic microglia c1b and c1c were preferentially located in the CTX (Fig. 4c and Extended Data Fig. 5f). Differentially expressed genes (DEGs) and GSEA in CD45^+^ cells from the CTX showed enrichment for Gene Ontology (GO) terms including chemotaxis (*CH25H*, *CX3CR1*, *CCL3*), regulation of cell death (*CEBPB*, *TLR4*, *TNF*) and neuron projection guidance (GO 0097485), whereas CD45^+^ cells from the GE were enriched for GO terms including antigen processing and presentation (*HLA-DRA*, *HLA-DPA1*, *HLA-DRB1*; GO 0019884), plasma lipoprotein assembly (*APOC1*, *APOC2*, *ABCA1*) and sister chromatid segregation (GO 0000819) (Fig. 4d,e and Supplementary Table 5). Microscopy combining antibodies and RNAscope probes for specific immune cell subtypes confirmed more abundant HLA^+^ cells (c6) and *CLDN5*^+^;*SLC2A 1*^+^;*IGFBP7*^+^;*MFSD2A*^+^;IBA1^+^ VAM (c4) inside the blood vessels in the GE than in the CTX (Fig. 4f,i and Extended Data Fig. 5g), whereas the number of *JAML*^+^;*LYZ*^+^;S100A9^+^ monocytes showed no regional differences (Fig. 4h,i). Collectively, these results provide the first comprehensive analysis of CD45^+^ immune cell subtypes and their interactions with blood vessels in cortical plates and GE of the prenatal human brain. Combined with IBA1 IEM data and live imaging data (Figs. 1h and 2a,b), this scRNA-seq dataset probably captures CD45^+^ immune cells from their intravascular states to transendothelial migration and, finally, becoming homeostatic microglia.

### Flow cytometry validates immune cell subtypes

**Fig. 5 |:**
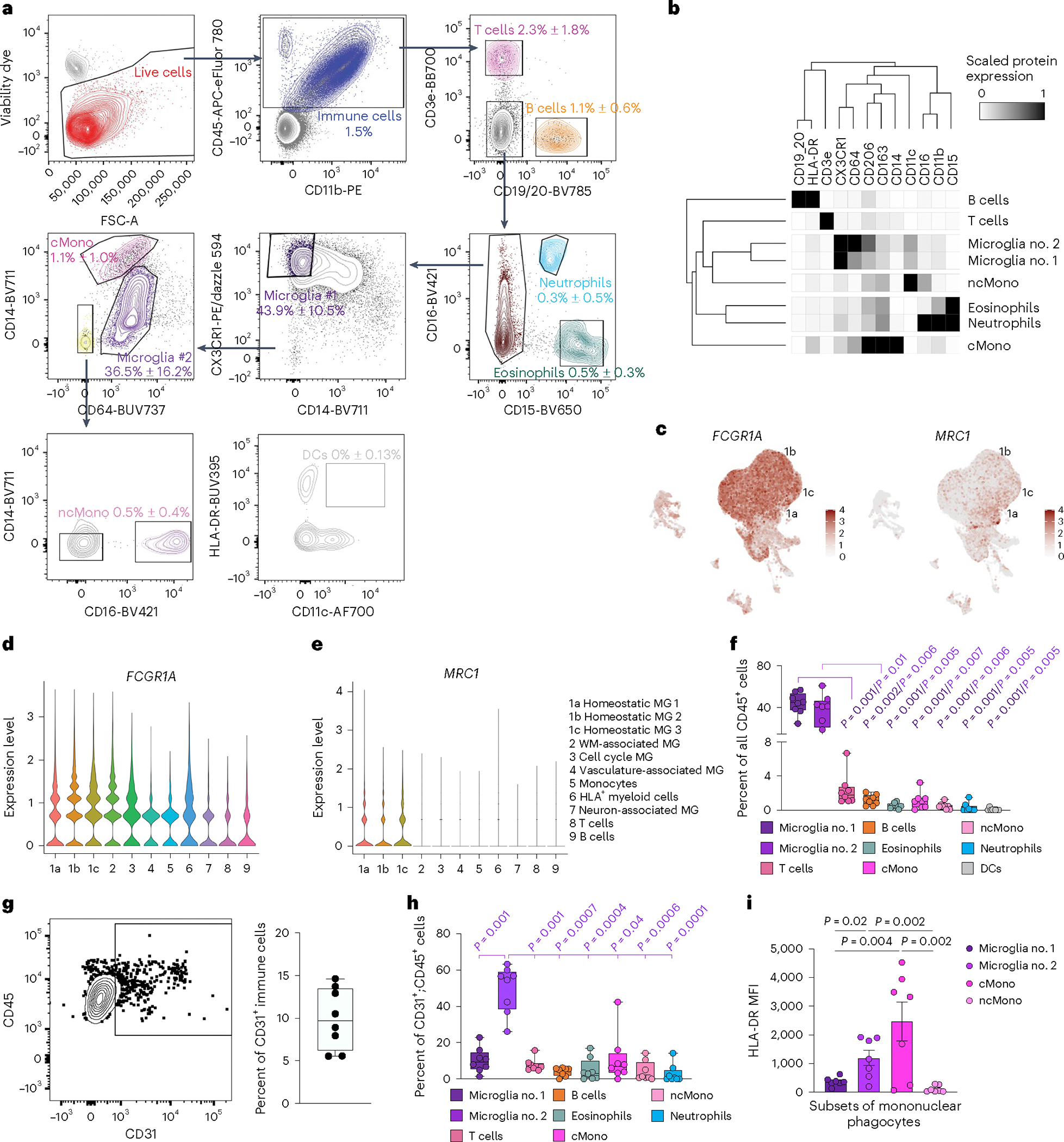
High-dimensional flow cytometry characterizes subtypes of CD45^+^ immune cells in prenatal human brain. **a**, Gating strategy used in high-dimensional flow cytometry to distinguish nine different immune cell types, including T cells, B cells, neutrophils, eosinophils, microglia, BAM, classical monocytes (cMono), non-classical monocytes (ncMono) and dendritic cells (DCs). Briefly, within single live CD45^+^ immune cells, CD3e and CD19/20 positively gate T cells and B cells, respectively. From CD3e^−^;CD19/20^−^ cells, CD15^+^;CD16^+^ cells are gated as neutrophils and CD15^+^;CD16^−^ cells are gated as eosinophils. Among CD15^−^ cells, microglia no. 1 are gated as CX3CR1^hi^;CD14^lo^. All the remaining cells that not designated as microglia no. 1 then proceed to the next gating, in which CD64^hi^;CD14^lo^ cells are gated as microglia no. 2, and CD64^lo^;CD14^hi^ cells are gated as cMono. Within CD64^−^;CD14^−^ cells, CD16^+^ cells are gated asn ncMono. CD11c and HLA-DR markers confirm that HLA-DR^+^ cells are not likely to be DCs. **b**, A heat map showing expression levels of cell surface markers in each immune cell subtype from high-dimensional flow cytometry. **c**–**e**, Feature plots (**c**) and violin plots showing the expression of *FCGR1A* (CD64) (**d**) and *MRC1* (CD206) (**e**) transcripts based on scRNA-seq data in Fig. 4a. MG, microglia; WM, white matter. **f**, Relative abundance of different immune cell subtypes among all CD45^+^ cells. **g**,**h**, Gating strategy for CD45^+^;CD31^+^ cells and the relative abundance of CD45^+^;CD31^+^ cells among all CD45^+^ cells. **i**, Mean fluorescence intensity (MFI) of HLA-DR in microglia no. 1, microglia no. 2, cMono and ncMono. Statistics in **i** use a one-way analysis of variance with a Mann–Whitney test, and the data represent the mean ± standard error of the mean. For the box and whisker plots in **f**–**h**, the center lines denote the median values (50th percentile), the boxes contain the 25–75th percentiles of the dataset, and the whiskers mark the minimal and maximal values. The data in **b** and **f**–**i** are from eight independent biological samples.

To further validate immune cell subtypes, we performed high-dimensional flow cytometry by applying 16 cell surface markers on CD45^+^ cells from the second trimester prenatal human brain. After gating for CD45^+^ immune cells, we identified nine immune cell subtypes, including two groups of microglia. The first group, microglia no. 1, was defined by CX3CR1^hi^;CD14^−^ expression, as previously described in human and mice^30,31^, and the second group, microglia no. 2, was defined by CD64^hi^;CD14^lo^ that included BAM based on relatively high expression of CD206 (ref. 16,17) (Fig. 5a,b). In addition, we identified classical monocytes (CD64^lo^;CD14^hi^), non-classical monocytes (CD64^−^CD14^−^;CD16^+^), dendritic cells (CD64^−^;CD14^−^;CD16^−^;HLA-DR^+^;CD 11c^+^), T cells, B cells, eosinophils and neutrophils (Fig. 5a,b). Similar to adult mouse brain^31^, several cell surface proteins identified in microglia and BAM, including CD64 (encoded by *FCGR1A*) and CD206 (encoded by *MRC1*), were expressed in homeostatic microglia clusters 1a, 1b and 1c in our scRNA-seq dataset (Fig. 5c–e). Based on flow cytometry data, microglia no. 1 and microglia no. 2 each represented ~40% of the entire CD45^+^ immune cells in prenatal human brain, whereas T cells and B cells each represented ~1–2% and monocytes and dendritic cells each represented less than 1% (Fig. 5f). To reconcile the VAM (c4) cluster from our scRNA-seq with results from high-dimensional cytometry, we showed that 5–15% of CD45^+^ cells coexpressed CD31, a canonical endothelial cell marker (Fig. 5g). Among CD45^+^;CD31^+^ cells, microglia no. 2 comprised ~50%, and only ~10% were classical monocytes or microglia no. 1 (Fig. 5h). Finally, we showed that classical monocytes expressed the highest levels of HLA-DR, followed by microglia no. 1 and no. 2 (Fig. 4j). Thus, the majority of HLA^+^ myeloid cells (c6) in our scRNA-seq dataset most likely consisted of classical monocytes and some microglia.

**Extended Data Fig. 6 |:**
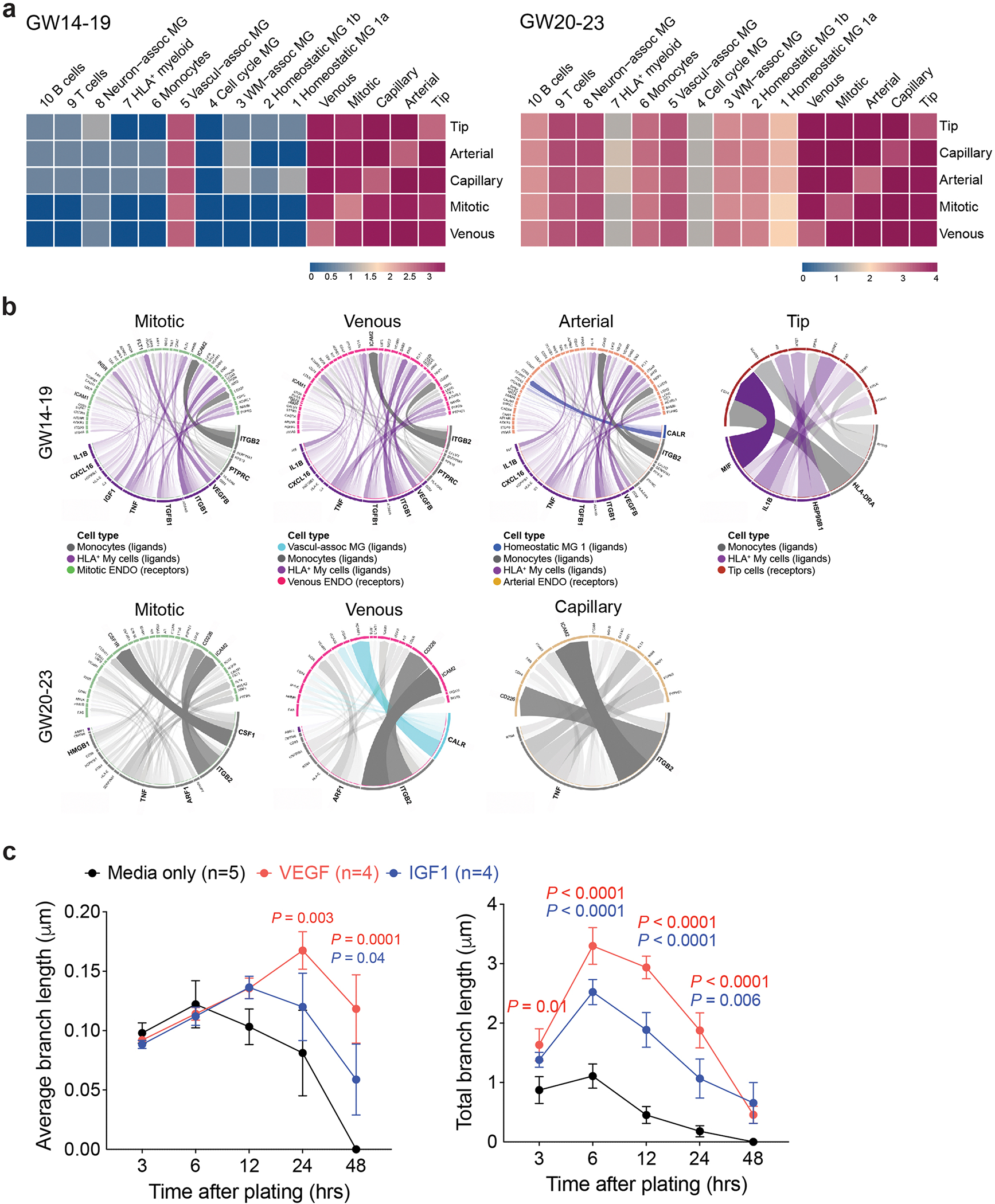
Bioinformatic analyses reveal potential signaling mechanisms that regulate the interactions between CD45^+^ immune cells and different subtypes of endothelial cells. (**a**) CellphoneDB analyses reveal stage-dependent communications via ligand-receptor pairs between CD45^+^ subtypes and CD31^+^ endothelial subtypes at GW14-19 and GW20-23. (**b**) NicheNet analyses predict signaling pathways used by homeostatic microglia (c1a), HLA^+^ myeloid cells, monocytes, and VAM to interact with endothelial subtypes at GW14-19 and GW20-23. Data from panels **a** and **b** are from 2 independent biological samples at GW14-19, and 3 independent biological samples at GW20-23. ENDO, endothelial cells. MG, microglia. (**c**) Quantification of average and total endothelial branch lengths formed by HUVEC in Matrigel-based assays. The conditions include EGM-2 media alone or addition of VEGF or IGF1, which is one of the ligands used by CD45^+^ cells identified by NicheNet. Statistics use two-tailed, unpaired Student’s t-test, data represent mean ± SEM. The P values represent comparisons between HUVECs treated with VEGF or IGF1 *vs* no treatment. Not significant comparisons are not shown. *n* indicates the number of independent biological samples used for quantification.

To understand how immune cells regulate angiogenesis in prenatal human brain, we used CellPhoneDB to predict cell–cell communication via ligand–receptor pairs between CD45^+^ cells and endothelial cells at the same gestational ages^4,32^. This approach showed that at GW14–19, all endothelial cell subtypes, including tip cells, mitotic, venous, arterial and capillary endothelial cells, had extensive cell–cell communications with one another^4^. However, only VAM (c4) among CD45^+^ cells exhibited cell–cell communication with endothelial cells (Extended Data Fig. 6a). By GW20–23, most CD45^+^ subtypes showed cell–cell communication with endothelial cells, consistent with the stage-dependent role of CD45^+^ cell to promote endothelial branch formation in Matrigel (Fig. 3d,e). Next, we used NicheNet to predict ligand–target gene links between these two cell types^33^, focusing on HLA^+^ myeloid cells (c6), monocytes, VAM and homeostatic microglia (c1a) in the GE. Our results showed that at GW14–19, both HLA^+^ myeloid cells and monocytes showed robust interactions with all endothelial cell subtypes (Extended Data Fig. 6b). By GW20–23, monocytes exhibited the most robust signaling pathways in interacting with endothelial cells in the GE. The most prominent ligand–target gene link was ITGB2 from monocytes and ICAM2 receptor from endothelial cells^34^. Additional ligands included VEGFB (Vascular Endothelial Growth Factor B), IGF1 (Insulin Like Growth Factor 1), TGFB1 (Transforming Growth Factor Beta 1), TNF (Tumor Necrosis Factor), CXCL16 and IL1B (Interleukin 1 Beta), among these VEGF (25 ng ml^−1^) and IGF1 (5 ng ml^−1^) increased endothelial cell branch lengths in Matrigel^35^ (Extended Data Fig. 6c).

### GMH harbors activated neutrophils and monocytes

**Extended Data Fig. 7 |:**
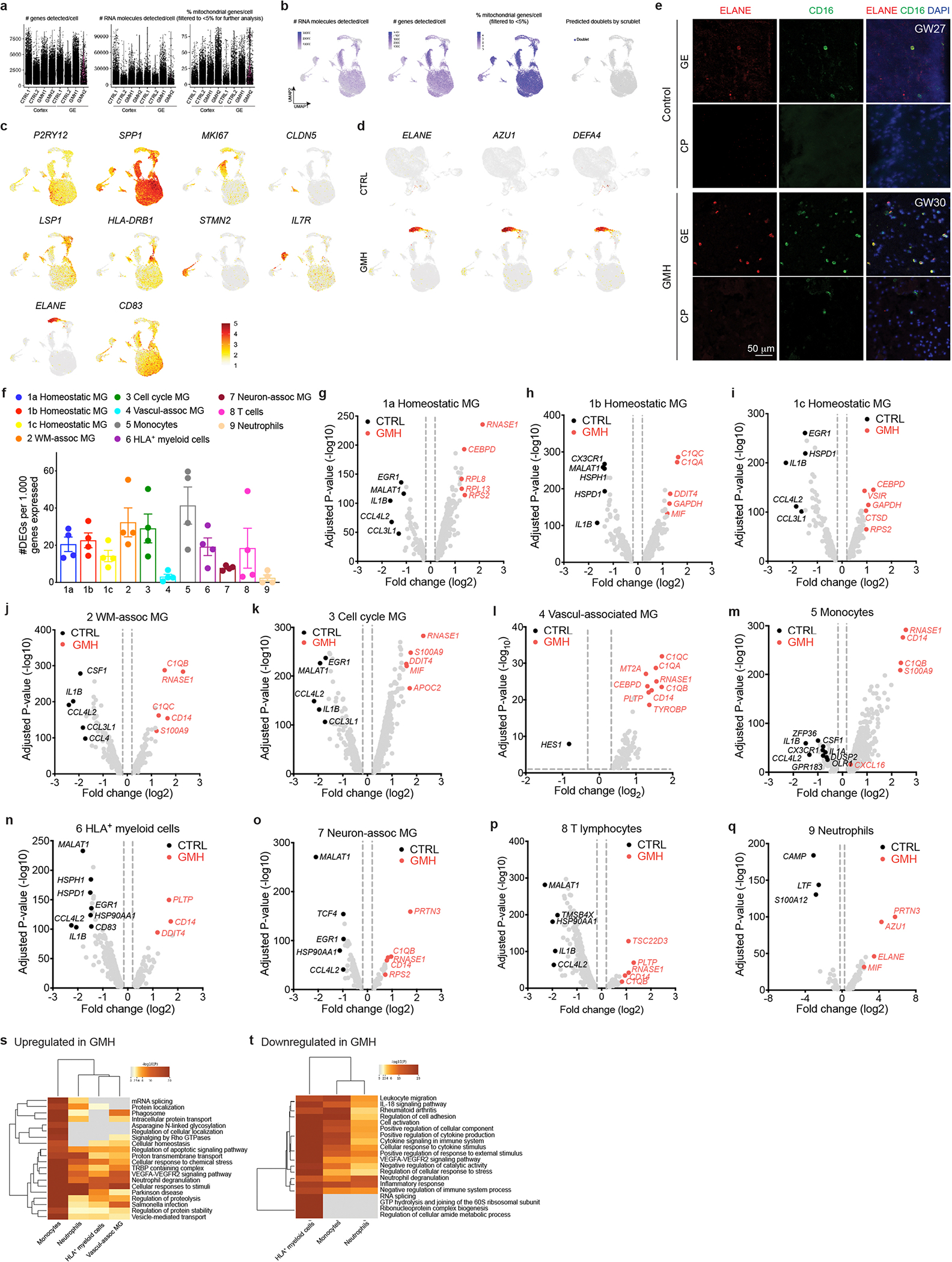
Single-cell transcriptomics in CD45^+^ cells from GMH cases reveal activation of proinflammatory transcriptomic profiles in monocytes, HLA^+^ myeloid cells, vasculature-associated microglia (VAM), neutrophils, and other immune cell types. (**a-b**) Quantifications of the number of genes, RNA molecules, and percentage of mitochondrial genes per cell, as well as predicted doublets from scRNA-seq in age-matched CTRL and GMH cases. Each dot represents one cell. (**c**) UMAP feature plots of marker gene expressions that define each subtype of CD45^+^ cells. (**d**) UMAP feature plots of neutrophil transcripts *ELANE*, *AZU1*, and *DEFA4* in CD45^+^ cells from CTRL or GMH cases. (**e**) Confocal images show higher abundance of ELANE^+^;CD16^+^ cells in the GE of GMH cases compared to age-matched controls. This experiment was repeated in same number of independent biological replicates for control and GMH cases as in Fig. 5e. (**f**) Gene burden analyses of the relative abundance of DEGs in all immune cell types. Data are from 2 independent biological samples in each condition (Control, GMH) and represent mean ± SEM. Each data point represents one pairwise comparision between a control and a GMH sample. (**g-q**) Volcano plots show differentially expressed genes from GMH *vs* CTRL cases in all CD45^+^ cell subtypes, including homeostatic microglia (MG), white matter-associated microglia (MG), cell cycle microglia (MG), vasculature-associated microglia (MG), monocytes, HLA^+^ myeloid cells, neuron-associated microglia (MG), T lymphocytes, and neutrophils. Adjusted P-values and fold changes were calculated from pseudo-bulked scRNA-seq data using DESeq2. By default in DESeq2, *P*-values attained by the Wald test are corrected for multiple testing using the Benjamini and Hochberg method. Genes shown were filtered to be below adjusted p-value of 0.05 and above fold change of 1.2 between CTRL and GMH comparisons. Vertical dashed lines indicate the boundary of significance in fold change. (**s-t**) Heatmap of GO terms in CD45^+^ cell subtypes highly associated with the GE vasculature, including monocytes, vasculature-associated microglia (MG), HLA^+^ myeloid cells, and neutrophils, which are upregulated (**s**) or downregulated (**t**) in GMH cases compared to age-matched controls. Data in all panels except **e** are from 2 independent biological samples in each condition (Control, GMH).

**Fig. 6 |:**
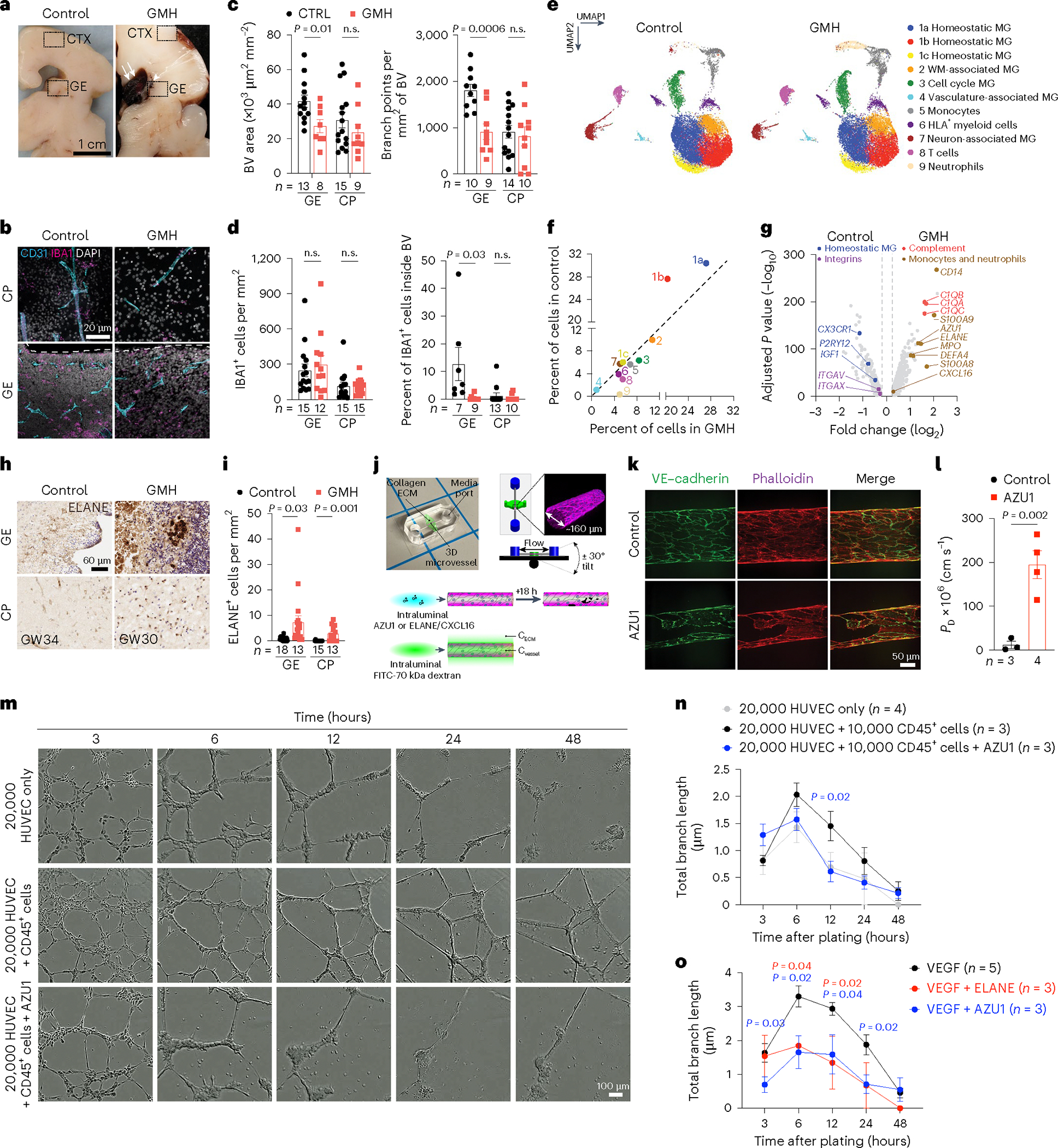
Single-cell transcriptomics in CD45^+^ cells from GMH cases reveal activated neutrophils. **a**, Gross images of a control prenatal brain (GW23) and a brain with GMH (GW24). **b**, IBA1^+^ cells and their relationship with CD31^+^ endothelial cells in the GE and cortical plate (CP) of control and GMH human brains. **c**, Quantification of densities of blood vessels and vascular branch points in the GE and CP of control and GMH cases. **d**, Quantification of IBA1^+^ cells and percentage of intravascular IBA1^+^ cells in the GE and CP of control and GMH cases. **e**, UMAP comparing CD45^+^ subtypes from GMH cases and age-matched control. MG, microglia; WM, white matter. **f**, A distribution plot comparing the relative abundance of CD45^+^ subtype in control versus GMH cases. **g**, A volcano plot showing DEGs and GO terms identified by pseudobulked scRNA-seq data in CD45^+^ cells from GMH versus control cases. The adjusted *P* values and fold changes were calculated using DESeq2. By default in DESeq2, the *P* values attained by the Wald test are corrected for multiple testing using the Benjamini–Hochberg method. The dashed lines indicate cutoffs for *P*value of 0.05 and fold change of 1.2. The data from **e**, **f** and **g** are from two independent biological samples in each condition (control, GMH). **h**–**i**, Immunohistochemical stain for ELANE show increased number of neutrophils in the GE and CP of GMH cases. **j**, Experimental setup of in vitro vascular permeeability assay using 3D microfluidic microvessels. **k**, Fluorescence micrographs of VE–cadherin and actin in control and AZU1-treated microvessels. **l**, Quantification of vascular permeability in control and AZU1-treated microvessels. **m**, Images of HUVEC in Matrigel-based branching morphogenesis. **n**,**o**, Quantification of average and total endothelial branch lengths formed by HUVEC in Matrigel-based assays, showing neutrophil proteins (AZU1, ELANE) can suppress CD45^+^ cell-mediated (**n**) or VEGF-mediated (**o**) vascular morphogenesis. Statistics in **c**, **d**, **i**, **l**, **n** and **o** use a two-tailed, unpaired Student’s *t*-test, and the data represent the mean ± standard error of the mean. n.s., not significant. In **n**, the *P* values represent comparisons between HUVEC coincubated with CD45^+^ cells versus HUVEC only. In **o**, the *P* values represent comparisons between VEGF-primed HUVEC treated with AZU1 or ELANE versus VEGF-primed HUVEC only.

To characterize how interactions between immune cell subtypes and vasculature could contribute to the pathogenesis of GMH, we found that vascular area and branch points were reduced in GE but not in the cortical plate of GMH cases (Fig. 6a–c). GMH cases also showed significantly lower percentage of IBA1^+^ cells inside the vascular lumen in the GE but not in the cortical plate (Fig. 6d). To investigate the composition of immune cells in GMH cases, we performed scRNA-seq on FACS-isolated CD45^+^ cells in the GE and CTX from two 24–25 GW GMH cases and two age-matched controls (Fig. 6e and Supplementary Table 1). After quality control, we obtained 35,275 cells with 42,052 reads and 2,098 genes per cell (Extended Data Fig. 7a,b). Clustering yielded 11 subtypes with similar marker genes as those found in the scRNA-seq dataset from control CD45^+^ cells (Fig. 6e, Extended Data Fig. 7c and Supplementary Table 6). Compared with the controls, GMH cases showed significant reduction in homeostatic microglia (c1a, c1b) but modest increases in cell cycle microglia (c3), monocytes (c5) and T cells (c8) (Fig. 6f). Furthermore, CD45^+^ cells in GMH cases contained a markedly expanded population of neutrophils (c9) that expressed high abundance of *ELANE*, *AZU1* and *DEFA4*, which encode for antimicrobial factors elastase, AZU1 and defensin alpha 4, respectively (Fig. 6e–g and Extended Data Fig. 7d). Neutrophils in GMH cases also showed increased expression of cell cycle gene *MKI67*, a well-established marker for activated neutrophils (Extended Data Fig. 7c)^36,37^. Immunostaining showed increases in ELANE^+^ cells in the GE and the cortical plate of GMH cases, many coexpressed CD16 (Fig. 6h,i and Extended Data Fig. 7e).

Pseudobulk analyses of DEG in CD45^+^ cells from GMH cases showed downregulation of homeostatic microglia genes (*CX3CR1*, *P2RY12*, *IGF1* and *SALL1*) and integrins (*ITGAV*and *ITGAX*) and upregulation of genes in the complement pathway (*C1QA*, *C1QB* and *C1QC*), monocytes (*CD14*, *S100A9* and *S100A8*), neutrophils (*AZU1*, *ELANE*, *MPO* and *DEFA4*) and chemokine *CXCL16* (Fig. 6g and Supplementary Table 7). Gene burden score and DEG analyses revealed transcriptomic changes existed in all CD45^+^ subtypes, including VAM, monocytes, HLA^+^ myeloid cells and neutrophils, as well as homeostatic microglia (Extended Data Fig. 7f–q). GO analysis of DEGs in VAM, monocytes, HLA^+^ myeloid cells and neutrophils from GMH cases revealed upregulation of cellular stress, neutrophil degranulation and phagosomes, as well as downregulation of leukocyte migration and cellular response to cytokine stimulus (Extended Data Fig. 7s,t and Supplementary Table 7).

### ELANE, CXCL16 and S1PR1 disrupt vascular integrity

**Fig. 7 |:**
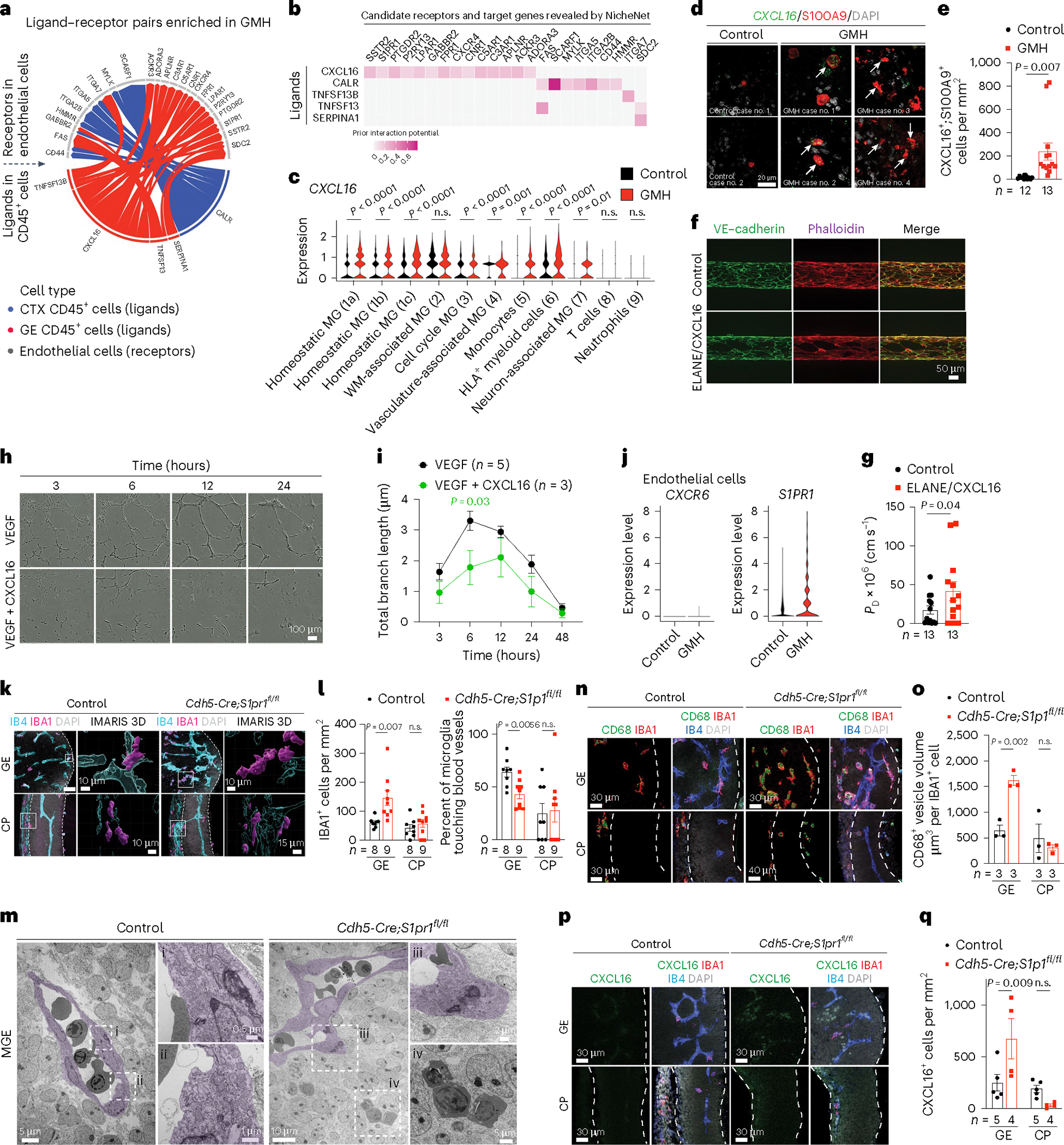
Dysregulated CXCL16^−^S1PR1 signaling disrupts angiogenesis in GE. **a**,**b**, A wheel plot (**a**) and heat map (**b**) from NicheNet analysis reveal ligand–receptor pairs between CD45^+^ immune cells and CD31^+^ endothelial cells dysregulated in GMH cases. The color intensity in the heat map indicates interaction potential (**b**). **c**, Violin plots show upregulated CXCL16 expression in most CD45^+^ subtypes in GMH cases. The data from **a**–**c** are from two independent biological samples in control and GMH cases. MG, microglia. WM, white matter. **d**,**e**, Images (**d**) and quantification (**e**) of *Cxcl16*^+^ and S100A9^+^ cells in GEs of control and GMH cases. Arrows in **d** indicate *CXCL16*^+^ and S100A9^+^ cells. **f**,**g**, Images (**f**) and quantification (**g**) of ELANE/CXCL16-treated microvessels show disorganization of VE–cadherin and actin and increased vascular permeability. **h**,**i**, Images (**h**) and quantification (**i**) of HUVEC branching morphogenesis treated with VEGF or VEGFand CXCL16. **j**, Violin plots show the expression of *CXCR6* and *S1PR1* in endothelial cells from control or GMH samples. **k**, Confocal and IMARIS 3D images of IBA1^+^ cells interacting with IB4^+^ vasculature in E12.5 control and *Cdh5*^*−*^*Cre;S1pr1*^*fl/fl*^ mice. White boxes indicate areas in the confocal images where enlarged IMARIS 3D images are captured. **l**, IBA1^+^ cell density and the percentage of IBA1^+^ cells touching blood vessels in the GE and cortical plate (CP) of E12.5 control and *Cdh5*^*−*^*Cre;S1pr1*^*fl/fl*^ mice. **m**, TEM shows myeloid cells inside the vascular lumen and in the brain parenchyma in the MGE of E12.5 control and *Cdh5*^*−*^*Cre;S1pr1*^*fl/fl*^ mice. The subdivisions (i–iv) show further magnification of TEM images. **n**,**o**, Confocal images and quantification show increased volume of CD68^+^ vesicles in IBA1^+^ cells in the GE of *Cdh5*^*−*^*Cre;S1pr1*^*fl/fl*^ mice (**n**) compared with the age-matched control (**o**). The dotted white lines define boundaries of the GE and CP where IBA1^+^ cells are quantified. **p**,**q**, Confocal images and quantification show increased density of CXCL16^+^ cells in the GE of *Cdh5*^*−*^*Cre;S1pr1*^*fl/fl*^ mice (**p**) compared with age-matched control (**q**). Statistics in **c**, **e**, **g**, **i**, **l**, **o** and **q** use a two-tailed, unpaired Student’s *t*-test, and the data represent the mean ± standard error of the mean. n.s., not significant. *n* indicates the number of independent biological samples used for quantification.

To investigate whether ELANE and AZU1 produced by the neutrophils may negatively impact angiogenesis in GMH^38^, we generated three-dimensional (3D) microvessels by seeding human microvascular endothelial cells (hMVECs) into a microfluidic channel surrounded by collagen (Fig. 6j)^39^. We then flowed AZU1 (100 μg ml^−1^) intraluminally for 18 h. This treatment significantly disrupted VE–cadherin complexes between endothelial cells and increased microvessel permeability, allowing fluorescently labeled 70 kDa dextran to leak into the interstitial matrix (Fig. 6k,l). Next, we showed that treatment with AZU1 or ELANE suppresses branching morphogenesis in HUVEC mediated by CD45^+^ cells or by VEGF (Fig. 6m–o). To characterize how the signaling pathways between immune and endothelial cells might contribute to hemorrhage in GMH cases, we used NicheNet to identify several ligands enriched in CD45^+^ cells from GMH cases that could promote vascular dysfunction (Fig. 7a–c). Among these, CXCL16-mediated signaling pathways have broad implications in inflammation under several disease conditions^40,41^. Indeed, expression of *CXCL16* was increased in several CD45^+^ subtypes, especially monocytes (Fig. 7c) and a significant increase in *CXCL16*^+^;S100A9^+^ monocytes in the GE of GMH cases, but not in control cases (Fig. 7d,e). Furthermore, similar to AZU1, CXCL16 and ELANE (both at 20 μg ml^−1^) disrupted vascular endothelial cadherin (VE–cadherin) junctional complexes and increased vascular permeability in 3D microvessels (Fig. 7f,g), and CXCL16 (10 μg ml^−1^) alone suppressed VEGF-mediated vascular morphogenesis in 3D Matrigel-based assays (Fig. 7h,i).

**Extended Data Fig. 8 |:**
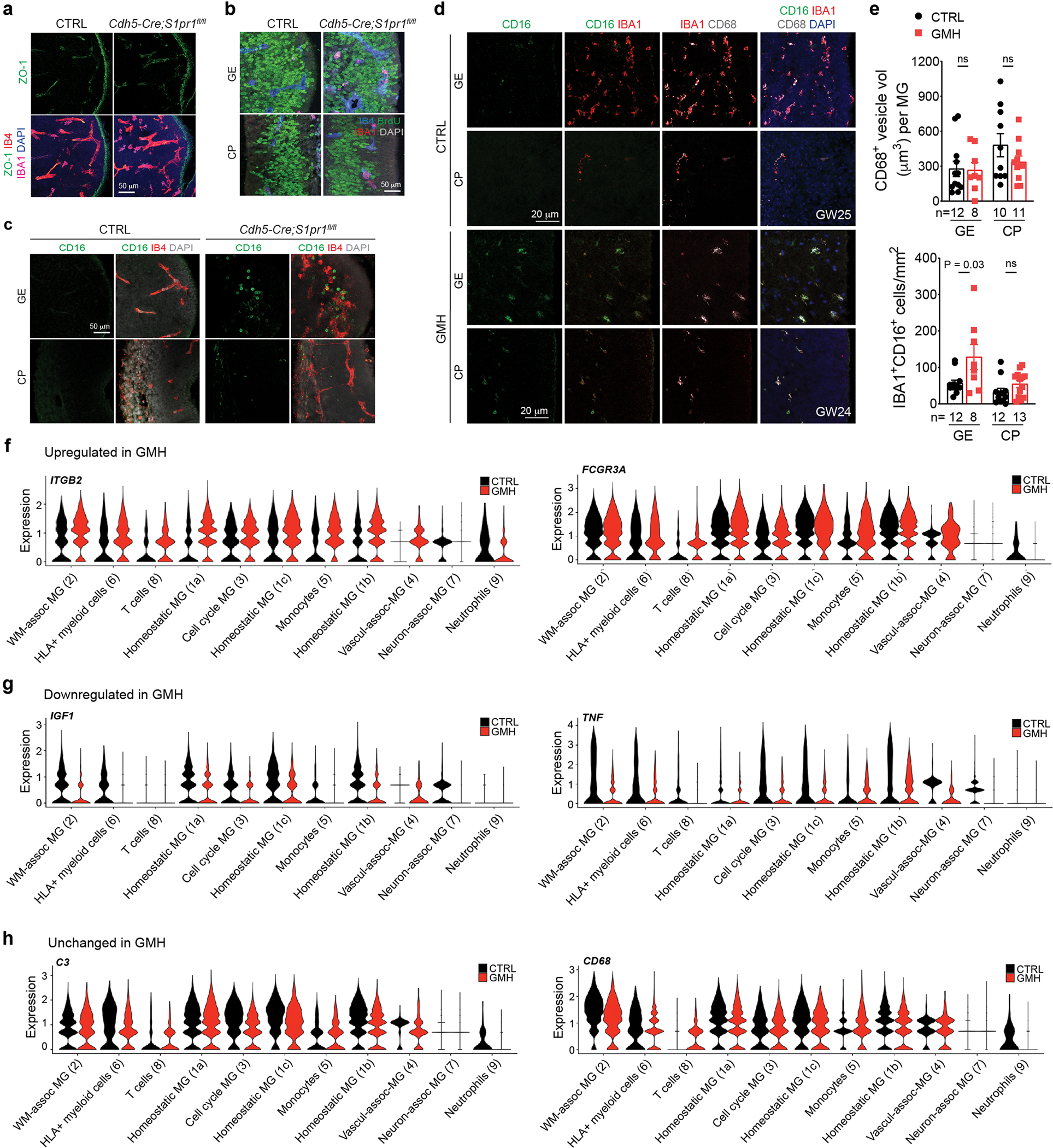
Dysregulated CXCL16-S1PR1 signaling disrupts angiogenesis in the GE. (**a**) Confocal images show expression of tight junction ZO-1 in the GE of E12.5 CTRL and *Cdh5*^*−*^*Cre;S1pr1*^*fl/fl*^ mice. (**b**) Confocal images show similar proliferation in IBA1^+^ cells in the GE and CP of CTRL and *Cdh5*^*−*^*Cre;S1pr1*^*fl/fl*^ mice. (**c–e**) Confocal images and quantification show higher abundance of CD16^+^ cells in the GE of E12.5 *Cdh5*^*−*^*Cre;S1pr1*^*fl/fl*^ mice (**c**) and in the GE of GW24-25 human GMH cases (**d, e**), when compared to age-matched controls. CD68^+^ vesicle volume in IBA1^+^ cells remains unchanged between age-matched CTRL and GMH cases. *n* indicates the number of independent biological samples used for quantification. (**f–h**) Violin plots show upregulated (**f**), downregulated (**g**), and unchanged expressions (**h**) of key protein transcripts in most CD45^+^ cell subtypes from GMH cases, when compared to age-matched CTRL cases. Statistics in panel **e** use two-tailed, unpaired Student’s t-test, data represent mean ± SEM. ns, not significant. Data in panels **f-h** are from 2 independent biological samples in each condition (Control, GMH).

Further search for CXCL16 signaling partners showed that CXCL16 receptor *CXCR6* was nearly undetectable in endothelial cells from control and GMH cases. However, *S1PR1* was differentially expressed by endothelial cells from GMH cases (Fig. 7j). Since the S1P gradient provides a spatial cue for the trafficking of immune cells and the localization of S1PR1 is dynamically regulated in vascular cells^42,43^, we hypothesized that dysregulated *S1PR1* signaling in endothelial cells from GMH cases could disrupt homeostatic interactions between immune cells and the nascent vasculature. To test this, we deleted *S1PR1* in endothelial cells using *Cdh5*^*−*^*Cre* (*Cdh5*^*−*^*Cre;S1pr1*^*fl/fl*^)^44^. At E12.5, *Cdh5*^*−*^*Cre;S1pr1*^*fl/fl*^ mice expressed tight junction protein ZO-1 (also known as tight junction protein 1 or TJP1) in their nascent vasculature and had comparable proliferation rates in IBA1^+^ cells as controls (Extended Data Fig. 8a,b). However, E12.5 *Cdh5*^*−*^*Cre;S1pr1*^*fl/fl*^ mice showed a significant increase in IBA1^+^ cells and decrease in the percentage of IBA1^+^ cells attached to the extravascular surface of the blood vessels in GE but not in the cortical plate (Fig. 7k,l). Similarly, transmission electron microscopy (TEM) showed that myeloid cells in the GE of E12.5 *Cdh5*^*−*^*Cre;S1pr1*^*fl/fl*^ mice appeared to have migrated across endothelial cells into the brain parenchyma (Fig. 7m(i–iv)). Furthermore, IBA1^+^ cells from *Cdh5*^*−*^*Cre;S1pr1*^*fl/fl*^ mice contained significantly more abundant CD68^+^ vesicles in GE but not in the cortical plate (Fig. 7n,o) and showed increased expression of CXCL16 and CD16, similar to findings in GMH cases (Fig. 7p–q and Extended Data Fig. 8c–e). Consistent with these results, many CD45^+^ subtypes in GMH upregulated inflammation-related genes *ITGB2* and *FCGR3A* (encodes CD16) and downregulated angiogenic factors *IGF1* and *TNF* (Extended Data Fig. 8f–h). These results support that loss of S1PR1 in endothelial cells can activate IBA1^+^ cells, thereby upregulating their CD16 and CXCL16 expression.

### Exposure to ELANE and CXCL16 promotes hemorrhage in GE

**Extended Data Fig. 9 |:**
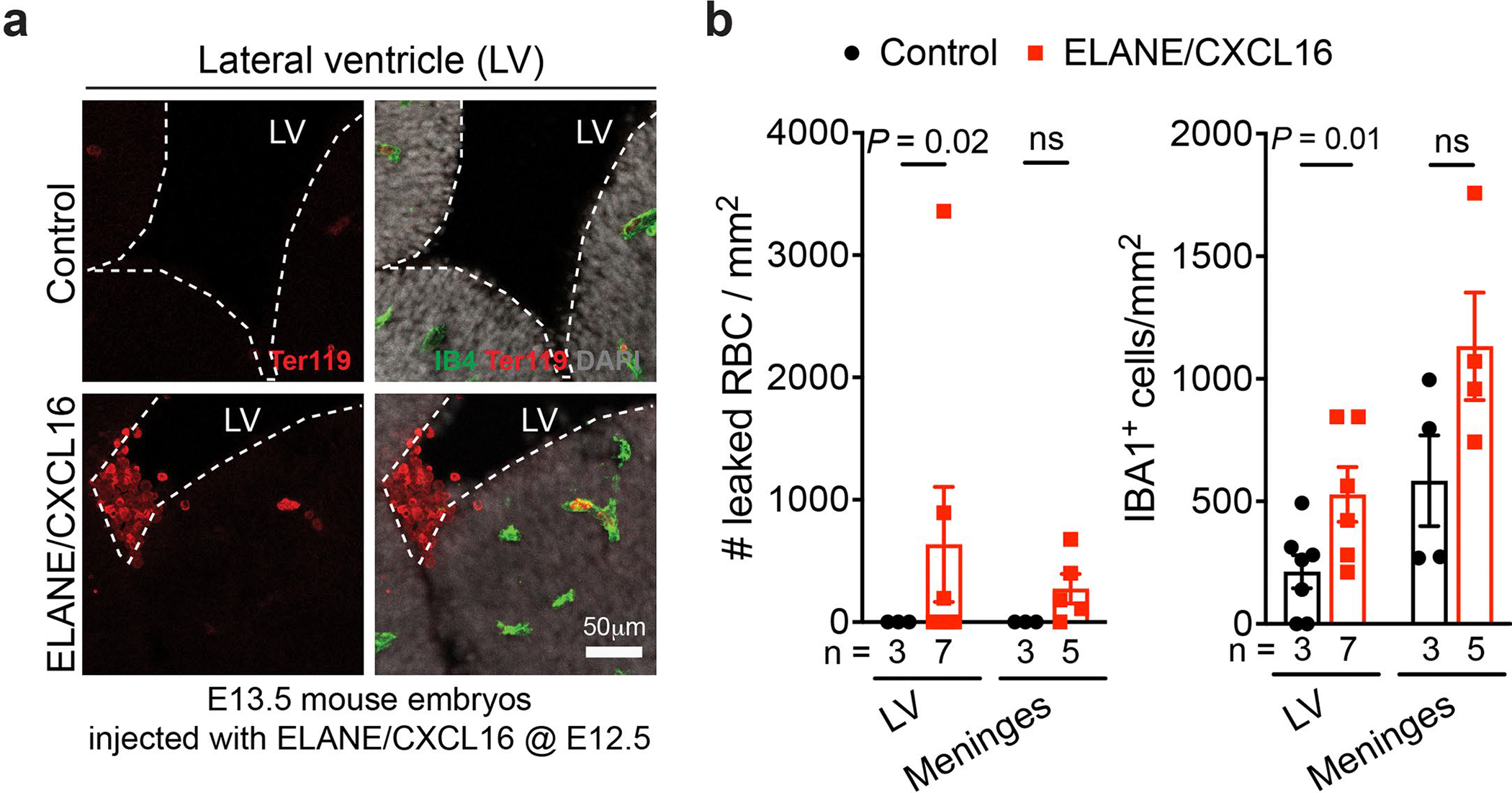
Exposure to proinflammatory factors ELANE and CXCL16 promotes intraventricular hemorrhage in embryonic mouse brain. (**a**) Confocal microscopic images show intraventricular hemorrhage, characterized by the presence of Ter119^+^ red blood cells (RBCs) in the lateral ventricle adjacent to the medial ganglionic eminence (MGE). LV, lateral ventricle, IB4, isolectin B4, a vascular marker. (**b**) Quantification shows significantly higher numbers of RBCs and IBA1^+^ cells in the lateral ventricle, and subtle increases of RBCs and IBA1^+^ cells in the meninges over the cortical plate. Statistics use two-tailed, unpaired Student’s t-test, data represent mean ± SEM. ns, not significant. *n* indicates the number of independent biological samples used for quantification.

**Fig. 8 |:**
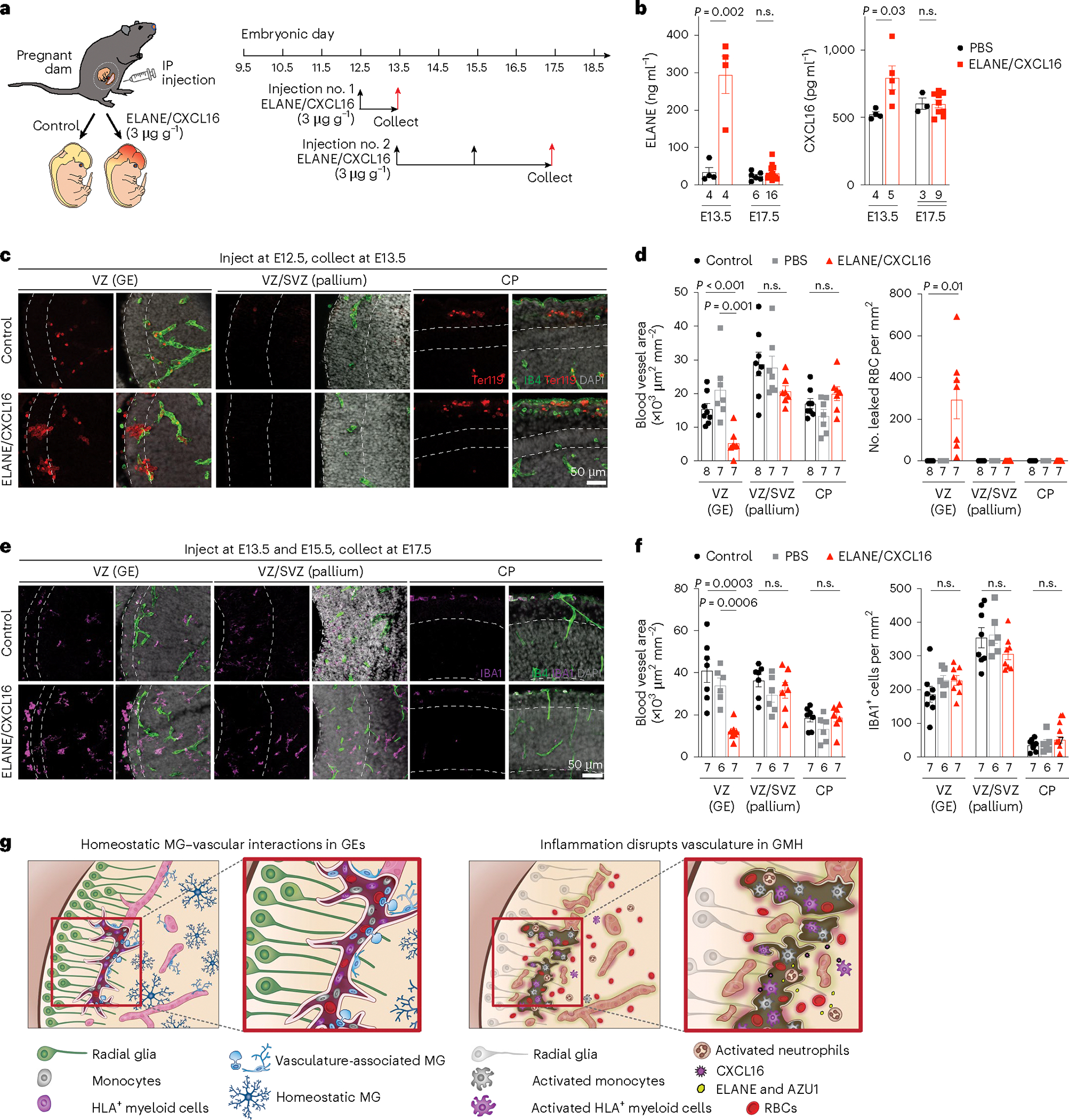
Proinflammatory factors ELANE and CXCL16 disrupt vasculature in GEs to promote hemorrhage in embryonic mouse brain. **a**, Schematic diagram showing two schedules of ELANE and CXCL16 intraperitoneal (IP) injection in pregnant mouse dams for embryonic brain tissue collection at E13.5 and E17.5. **b**, ELANE and CXCL16 protein concentrations in the plasma of PBS-injected control and ELANE/CXCL16-injected pregnant dams. **c**, Immunostaining with IB4 and Ter119 show leaked red blood cells in the GE but not in VZ/SVZ of the pallium or cortical plate (CP) of control or ELANE/CXCL16-injected embryos at E13.5. **d**, Quantifications of densities of blood vessels and leaked red blood cells (RBC) in VZ of the GE, VZ/SVZ of the pallium and CP in control of PBS-injected or ELANE/CXCL16-injected embryos at E13.5. **e**, Immunostaining with IB4 and IBA1 show reduced vascular density and increased ameboid morphology in IBA1^+^ cells in VZ of the GE but not in VZ/SVZ of the pallium or CP in ELANE/CXCL16-injected embryos at E17.5. **f**, Quantifications of densities of blood vessels and IBA1^+^ cells in VZ of the GE, VZ/SVZ of the pallium and CP in non-injected control of PBS-injected or ELANE/CXCL16-injected embryos at E17.5. Statistics in **b**, **d** and **f** use a two-tailed, unpaired Student’s *t*-test; n.s., not significant. *n* indicates the number of independent biological samples used for quantification. **g**, Left: schematic diagram depicting how subsets of CD45^+^ immune cells, including monocytes (gray), HLA^+^ myeloid cells (purple) and VAM (light blue) interact with the nascent vasculature to promote angiogenesis in the germinal matrix during the second trimester in prenatal human brain. Right: activated neutrophils produce bactericidal factors, such as ELANE and AZU1, whereas activated monocytes produce CXCL16 to create a proinflammatory milieu that disrupts nascent vasculature and promotes GMH. MG, microglia.

To examine the role of neutrophil- and monocyte-derived factors in vivo, we intraperitoneally injected wild-type timed-pregnant dams with ELANE and CXCL16 (each at 3 μg g^−1^body weight or ~40 μg ml^−1^) at E12.5 and collected embryos at E13.5 or injected at E13.5 and E15.5 and collected embryos at E17.5 (Fig. 8a). ELISA assays showed significantly higher concentration of ELANE and CXCL16 in ELANE/CXCL16-treated E13.5 pregnant dams but no detectable increase in ELANE/CXCL16-treated E17.5 pregnant dams (Fig. 8b). Histopathological examinations revealed that injection of ELANE/CXCL16 at E12.5 significantly reduced vascular area and disrupted vascular integrity, resulting in the accumulation of Ter119^+^ red blood cells in VZ of GE at E13.5 but not in VZ/SVZ of the pallium or in the cortical plate (Fig. 8c,d). Three out of seven ELANE/CXCL16-treated E13.5 embryos showed intraventricular hemorrhage (Extended Data Fig. 9a,b). Although E17.5 embryos from pregnant dams injected with ELANE/CXCL16 did not show obvious hemorrhage, they exhibited reduced vascular areas in the VZ of GE but not in the VZ/SVZ of the pallium or in the cortical plate (Fig. 8e,f and Extended Data Fig. 9a,b). In addition, IBA1^+^ cells in these embryos exhibited ameboid morphology in VZ of GE and lateral ventricle but no definitive increase in IBA1^+^ cell density (Fig. 8e,f). Together, these results support that a proinflammatory factors produced by activated immune cells can indeed damage the nascent vasculature and facilitate hemorrhage in GEs (Fig. 8g).

## Discussion

Our results uncover several key mechanisms on how the brain’s immune cells regulate angiogenesis in the prenatal brain. First, in prenatal mouse and human brain, nascent vasculatures in the GEs exhibit complex branching morphogenesis and are associated with more IBA1^+^ microglia and macrophages (Fig. 1 and Extended Data Figs. 1 and 2). Interestingly, ablation of IBA1^+^ cells in the embryonic mouse brain shows that these immune cells have region-specific and age-dependent role in promoting the formation of a complex vascular network in the GE but not in the cortical plate or in the VZ/SVZ of pallium (Fig. 2 and Extended Data Fig. 3). Consistent with these results, CD45^+^ cells from GW20^−^23 human brain exhibit transcriptomic features that are proangiogenic and are indeed more effective in promoting vascular morphogenesis in HUVEC cells than those from GW14–19 (Fig. 3). scRNA-seq and high-dimensional cytometry analyses of CD45^+^ cells from GW14–23 human brain further reveal specific CD45^+^ subtypes, including HLA^+^ myeloid cells, monocytes and VAM and how these cells utilize diverse signaling pathways to promote angiogenesis (Figs. 4 and 5 and Extended Data Figs. 5 and 6). Compared with previously published datasets from prenatal human and mouse brain^24,25,29^, our results not only capture the developmental trajectory of CD45^+^ cells, but they also provide insights into the mechanisms employed by subsets of CD45^+^ cells to promote angiogenesis in the GEs.

Our results uncover at least two mechanisms by which perturbations to immune–endothelial cell interactions contribute to GMH. First, activated neutrophils produce bactericidal factors, including ELANE and AZU1, that can disrupt the integrity of nascent vasculature in the GE of GMH cases (Fig. 6). Furthermore, AZU1 can disrupt vascular barrier permeability in microvessels as well as VEGF^−^ and CD45^+^ cell-mediated vascular morphogenesis (Fig. 6j–o). Second, aside from the bactericidal factors produced by activated neutrophils, many subtypes of CD45^+^ cells exhibit prominent transcriptomic changes suggesting their immune activation. Among signaling pathways identified in activated CD45^+^ subtypes, upregulation of CXCL16 in CD45^+^ cells, especially in monocytes (Figs. 6g and 7d,e), probably disrupts vascular integrity^40^. Indeed, intraperitoneal injection of ELANE and CXCL16 in pregnant dams at E12.5 disrupts vascular integrity and leads to hemorrhage in GE and lateral ventricle but not in the VZ/SVZ of the pallium or in the cortical plate at E13.5 (Fig. 8 and Extended Data Fig. 9). In contrast, injecting pregnant dams with ELANE and CXCL16 at E13.5 and E15.5 reduces vascular density in VZ of the GE at E17.5 without causing apparent hemorrhage (Fig. 8e,f).

Finally, our results show that the expression of S1PR1 is upregulated in endothelial cells from GMH cases (Fig. 7j), which most likely disrupts the S1P gradient required for the migration of immune cells across vascular barriers^42,43^. Indeed, endothelial cell-specific knockout of S1PR1 in embryonic mice leads to prominent infiltration of peripheral immune cells, including many that express CD68 and CXCL16 (Fig. 7n–q). These results suggest that CXCL16 and S1PR1 may constitute reciprocal interactions between immune cells and endothelial cells. As such, upregulation of CXCL16 in subsets of CD45^+^ cells and disruption of S1PR1 in endothelial cells could propagate a vicious cycle that exacerbates the egress of activated peripheral immune cells across the primitive BBB in GMH.

Despite the robust evidence that immune activation in preterm infants could contribute to the pathogenesis of GMH, there are several caveats to our study. First, our recent study shows that during the second trimester, nascent vasculatures in the GEs contain an ensemble of immature endothelial and mural cells that exhibit dynamic transcriptomic and metabolic profiles^4^. It is possible that these vascular cells, when challenged with exogenous insults, such as hypoxia and/or activated immune cells, may exhibit selective vulnerability to exacerbate hemorrhage. Second, given the intricate interplay among immune cells, vascular cells and neural stem/progenitor cells, it is possible that infection, inflammation and/or hypoxic–ischemic injury could disrupt this tripartite relationship and negatively impact the homeostatic interactions between angiogenesis and neurogenesis in GEs. Finally, given the high prevalence of comorbidities in preterm infants^45–47^, the causes for GMH in these patients are likely to be multifactorial. Indeed, our study shows that preterm infants with GMH exhibit a wide range of clinical features with variable lengths of postnatal survival (Supplementary Table 1) and several with low number of ELANE^+^ neutrophils close to those in age-matched controls (Fig. 6h,i). Future studies are needed to identify additional factors that could also contribute to the pathogenesis of GMH in these cases.

### Online content

Any methods, additional references, Nature Portfolio reporting summaries, source data, extended data, supplementary information, acknowledgements, peer review information; details of author contributions and competing interests; and statements of data and code availability are available at https://doi.org/10.1038/s41593-024-01769-2.

## Methods

### Human tissue collection

Deidentified age-matched control cases (*n* = 29) and cases with GMH (*n* = 16) of both sexes were collected from the Autopsy Service in the Department of Pathology at the University of California, San Francisco (UCSF) and La Fe Biobank (Supplementary Table 1) with previous patient consent in strict observance of the legal and institutional ethical regulations. The autopsy consent and all protocols for human prenatal brain tissue procurement were approved by the Human Gamete, Embryo and Stem Cell Research Committee (Institutional Review Board GESCR no. 10-02693) at the UCSF and by the University of California, San Diego Institutional Review Board (IRB 171379). All cases received diagnostic evaluations by a board-certified neuropathologist to be control or GMH. For immunohistochemistry and RNAscope, tissues were fixed with 4% paraformaldehyde (PFA) for 2 days, cryoprotected in a 30% sucrose gradient, embedded in optimal cutting temperature (OCT) media and cut at 30 μm with a Leica cryostat and mounted onto glass slides.

### Animals

#### Mice.

All experiments were conducted in accordance with the UCSF Institutional Animal Care and Use Committee guidelines (protocol no. AN169548). Mouse husbandry conditions, including ambient temperature, humidity and dark/light cycle followed the guidelines established by UCSF LARC (Laboratory Animal Resource Center). Mice carrying deletion of exon 5 of the mouse colony stimulating factor 1 receptor gene (*Csf1r*^+/*−*^) were obtained from the Jackson Laboratories (*B6.Cg-Csf1r*^*tm1.1Jwp*^*/J*, JAX #028064). Timed-pregnant mice (E12.5) were bred using female CD1 animals (Charles River Laboratories) and male *Cx3cr1*^*GFP*^ (B6.129P2(Cg)-*Cx3cr1*^*tm1Litt*^/J, JAX #005582) to visualize macrophages with green flourescent protein (GFP) in two-photon live imaging. E12.5 *Cdh5-Cre/+;S1pr1*^*fl/fl*^ mice and age-matched control littermates were provided by Dr. Julieta Alfonso (DKFZ). For histology, mice were perfused with 4% PFA before extraction of their brains, fixed in 4% PFA for 24 h, cryoprotected with 30% sucrose, embedded in OCT and sectioned at 20 μm coronally with a cryostat and mounted onto glass slides.

#### PLX5622 injections.

Colony stimulating factor 1 receptor (CSF1R)-antagonist, PLX5622 (Plexxikon) was dissolved in DMSO:PEG400: KolliphorRH40:PBS. Wild-type pregnant dams were intraperitoneal injected with 250 μl PLX5622 solution (50 mg kg^−1^) daily for seven consecutive days starting at E6.5, E10.5 or E12.5. Mouse embryos were collected 1 day after the final injection at E13.5, E17.5 or P0 for histological analyses.

### Antibodies

#### Primary antibodies.

The primary antibodies used in the procedure include: mouse anti-CD31 (DAKO, M082329-2, 1:200), sheep anti-CD31 (R&D Systems, AF806, 1:250), rabbit anti-IBA1 (FUJIFILM Wako Shibayagi, 019-19741, 1:3,000), goat anti-IBA1 (Novus Biological, NB100-1028, 1:250), fluorescein-labeled isolectin B4 (Vector Laboratories, FL-1201, 1:50), rabbit anti-ZO-1 (Thermo Fisher Scientific, 40-2200, 1:100), rat anti-BrdU (Abcam, ab6326, 1:500), rabbit anti-S100A9 (Abcam, ab63818, 1:500), mouse anti-HLA-DR + DP + DQ (Abcam, ab7856, 1:200), rabbit anti-ELANE (Abcam, ab131260, 1:1,000), mouse anti-CD16 (Santa Cruz Biotechnology, sc-20052, 1:100), rat anti-CD68 (Bio-Rad, MCA1957, 1:3,000), goat anti-CXCL16 (Thermo Fisher Scientific, PA5–47977, 1:50) and mouse anti-VE–cadherin (Santa Cruz Biotechnology, sc-9989, 1:300).

#### Secondary antibodies.

The secondary antibodies used in the procedure include: donkey anti-mouse Alexa Fluor 488 (Thermo Fisher Scientific, A-21202, 1:300), donkey anti-sheep Alexa Fluor 488 (Thermo Fisher Scientific, A-11015, 1:300), donkey anti-mouse Alexa Fluor 568 (Thermo Fisher Scientific, A-10037, 1:300), donkey anti-rat Alexa fluor 594 (Thermo Fisher Scientific, A-21209, 1:300), donkey anti-mouse Alexa Fluor 647 (Thermo Fisher Scientific, A-31571, 1:300), donkey anti-rabbit Alexa Fluor 647 (Thermo Fisher Scientific, A-31573, 1:300), donkey anti-goat Alexa Fluor 647 (Thermo Fisher Scientific, A-21447, 1:300) and goat anti-mouse Alexa Fluor 647 (Thermo Fisher Scientific, A-21236, 1:300).

#### Conjugated antibodies.

The conjugated antibodies used in the procedure include: mouse anti-CD45-PECy7 (BD Biosciences, 557748, 1:200), mouse anti-CD11b-FITC (Thermo Fisher Scientific, 11-0112-41, 1:200), mouse anti-CD16-PerCP Cy5.5 (BD Biosciences, 560717, 1:200), mouse anti-CD14-APC (BD Biosciences, 561708, 1:200), mouse anti-CD141(BDCA-3)-FITC (Miltenyi Biotec, 130–113-321, 1:40), mouse anti-CD3-BB700 (BD Biosciences, 566575, 1:40), mouse anti-CD11b(M1/70)-PE (Invitrogen, 12-0112-82, 1:40), rat anti-CX3CR1-PE/Dazzle 594 (BioLegend, 341624, 1:20), mouse anti-CD1c-PE/Cyanine7 (BioLegend, 331516, 1:40), mouse anti-CD163-Alexa Fluor 647 (BioLegend, 333620, 1:40), Mouse anti-CD11c(3.9)-Alexa Fluor 700 (Invitrogen, 56-0116-42, 1:10), mouse anti-CD45(HI30)-APC-eFluor 780 (Invitrogen, 47-0459-42, 1:40), mouse anti-CD16-Brilliant Violet 421 (BioLegend, 302037, 1:40), mouse anti-CD31-Brilliant Violet 605 (BioLegend, 303122, 1:40), mouse anti-CD15(SSEA-1)-Brilliant Violet 650 (BioLegend, 323034, 1:40), mouse anti-CD14-Brilliant Violet 711 (BioLegend, 301838, 1:40), mouse anti-CD19-Brilliant Violet 785 (BioLegend, 302240, 1:40), mouse anti-CD20-Brilliant Violet 785 (BioLegend, 302356, 1:40), mouse anti-HLA-DR-BUV395 (BD Biosciences, 564040, 1:40), mouse anti-CD64-BUV737 (BD Biosciences, 564425, 1:40), PECy7 mouse IgG1, κ isotype control (BD Biosciences, 557872, 1:200), Alexa Fluor 488 mouse IgG2a, κ isotype control (BD Biosciences, 557703, 1:200), PECy7 mouse IgG1, κ isotype control (BD Biosciences, 347202, 1:200) and APC mouse IgG1, κ isotype control (BD Biosciences, 555751, 1:200).

### Immunohistochemical staining

Chromogenic immunohistochemistr y method using 3,3′-diaminobenzidine (DAB) was performed on our samples. The slides were equilibrated at room temperature for 2 h and baked at 60 °C for 30 min. The sections were rinsed in Tris buffered saline (TBS) and quenched with 10% methanol and 3% H_2_0_2_ in TBS for 10 min. Antigen retrieval was performed using 0.01 M sodium citrate buffer (pH 6.0) for 10 min at 95 °C. The sections were rinsed three times with TBS and incubated with TBS+ (10% goat serum and 0.2% Triton X-100) blocking buffer for 1 h, followed by overnight incubation of primary antibodies at room temperature. Biotinylated secondaries were incubated for 2 h at room temperature and rinsed three times with TBS. The VECTASTAIN Elite ABC horse radish peroxidase (HRP) system with A (Avidin) and B (biotinylated HRP) complexes in TBS++++ (10% goat serum, 3% bovine serum albumin (BSA), 1% glycine and 0.4% Triton X-100) was used to amplify staining specificity. The signals were developed in DAB reaction solution (0.05% DAB and 0.05% H_2_0_2_ in 0.1 M Tris, pH 8.0) and stopped with three washes in 0.1 M Tris (pH 8.0), ending with dehydration in 100% ethanol and counterstaining using Nissl.

### Immunofluorescent staining

Tissue-mounted slides were defrosted overnight at 4 °C and then equilibrated to room temperature for 3 h. Antigen retrieval was performed on selected antigens with 10 mM sodium citrate buffer (pH 6.0) at 95 °C for 10 min. The samples were then washed with TBS for 5 min and repeated three times, before blocking with TBS++++ for 1 h. The slides were incubated with primary antibodies with denoted dilutions overnight at room temperature. Alexa fluorophore-conjugated secondary antibodies diluted in TBS++++ (1:300) were added on the following day for 2 h. The slides were then stained with DAPI and coverslipped.

### RNAscope

Human-specific probes (*JAML*, *LYZ*, *SLC2A1*, *CLDN5*, *SPP1*, *IGFBP7*, *MFSD2A* and *CXCL16*) were obtained from Advanced Cell Diagnostics (ACD). The slides were taken from −80 °C, dried at 60 °C for 1 h and fixed in 4% PFA for 2 h. The sections were then washed with PBS and treated with H_2_0_2_ (ACD) for 10 min at room temperature. All sections were treated with target retrieval buffer (ACD) for 5 min at 98–100 °C, before dehydration with 100% ethanol and baking at 60 °C for 30 min. The sections were left to dry overnight at room temperature. The following day, the sections were treated with protease III (ACD) for 15 min at 40 °C in the RNAscope hybridization oven before probe hybridization and amplification. All the following steps at 40 °C were conducted in the RNAscope hybridization oven oven. For hybridization, the sections were incubated with the desired probes for 2 h at 40 °C, and then rinsed in 1× wash buffer (ACD) twice for 2 min each. For amplification, sections were incubated with Amp 1 (ACD) for 30 min at 40 °C and then rinsed twice in the wash buffer for 2 min each. This was repeated with Amp 2 (ACD) for 30 min and Amp 3 (ACD) for 15 min at 40 °C, with wash buffer rinsing between the incubations. HRP-C1 (ACD) was then added for 15 min, followed by Opal Dye (1:100) for 30 min and, finally, a 15 min incubation with HRP blocker (ACD), all performed at 40 °C. In between all these steps, sections were rinsed twice for 2 min with 1× wash buffer. HRP signal, Opal Dye and HRP blocker steps were repeated for C2 and C3 probes. This was followed by the immunofluorescent staining protocol outlined above without the antigen retrieval step.

### Tissue clearing and immunofluorescent staining

The samples were optically cleared and immunostained following previously published SHIELD and iDISCO protocols^48,49^. Some 1–2-mm-thick coronal sections were cut from 0.5% PFA-fixed samples and incubated in 25% SHIELD-buffer solution and 50% SHIELD-epoxy solution (both from Lifecanvas Technologies) at 4 °C with gentle shaking for 2 days. This was followed by an incubation with a 1:1 ratio of SHIELD-ON buffer (Lifecanvas Technologies) and SHIELD-epoxy solution at 20 °C with gentle shaking for 1 day. The sections were then passively cleared with an SDS-based solution (300 mM sodium dodecyl sulfate, 10 mM boric acid and 100 mM sodium sulfite titrated to pH 9) at 55–60 °C with gentle shaking until tissue was transparent. After clearing, the samples were immunostained following iDISCO-based steps. Briefly, sections were incubated in permeabilization solution (20% dimethyl sulfoxide (DMSO), 0.16% Triton X-100 and 23 mg ml^−1^glycine in PBS) and then blocking solution (10% DMSO, 5% donkey serum and 0.168% Triton X-100 in PBS) at 37 °C for 2 days in each solution. The sections were then incubated with primary antibodies in PTwH (0.2% Tween-20 and 0.01 mg ml^−1^heparin in PBS) with 5% DMSO and 3% donkey serum and secondary antibodies in PTwH with 3% donkey serum at 37 °C for 7 days in each antibody step with gentle shaking. The sections were washed in PTwH at room temperature for a day between the two antibody incubation steps. Finally, the sections were refractive index-matched in EasyIndex (Lifecanvas Technologies) overnight at room temperature before imaging.

### Agarose embedding and light-sheet imaging

Refractive index-matched sections were embedded in 1.8% low melting-point agarose (Thermo Fisher) and emerged in EasyIndex (Lifecanvas Technologies) on custom made sample holders. The samples were imaged using an axially swept light-sheet microscope (SmartSPIM, Lifecanvas Technologies) equipped with a 3.6×, numerical aperture (NA) 0.2 detection objective (uniform axial resolution 3.2–4.0 μm) and a 2,048 × 2,048 sCMOS camera.

### Confocal imaging, processing and quantifications

**Extended Data Fig. 10 |:**
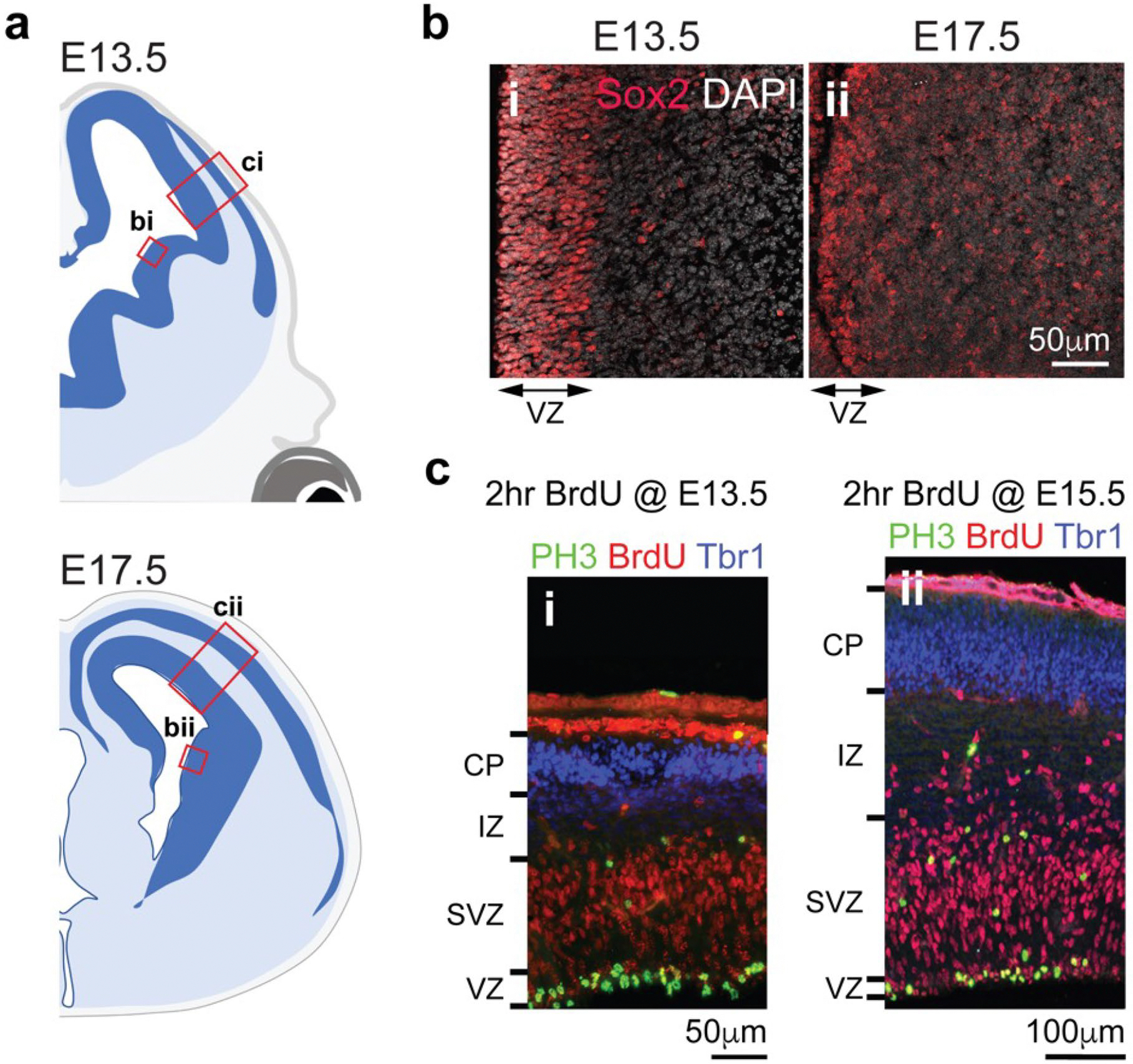
Confocal imaging, processing and quantifications of neural progenitors in GE and cortical plate. (**a, b**) We defined the ventricular zone (VZ) in ganglionic eminences (GE) in E13.5 and E17.5 mouse brain using the expression of Sox2, which delineates an active neurogenic niche with a layer of proliferative neural progenitors. (**c**) For the pallium, we used phospho-histone 3 (PH3) and BrdU (2 hr injection paradigm) to define the ventricular zone/subventricular zone (VZ/SVZ) and cortical neuron marker Tbr1 to define the cortical plate.

Confocal images were acquired on a Leica TCS SP8 confocal microscope using a 63× (NA 1.4) objective. We defined the VZ in GEs in E13.5 and E17.5 mouse brain using the expression of Sox2, which delineates an active neurogenic niche with a layer of proliferative neural progenitors^50^ (Extended Data Fig. 10a,b). For the pallium, we used phospho-histone 3 and BrdU (2 h injection paradigm) to define the VZ/SVZ and cortical neuron marker Tbr1 to define the cortical plate (Extended Data Fig. 10a,c). The delineation of these structures was further assisted using Nissl-stained images in atlases of prenatal mouse brain^51,52^. The images were processed and quantified using ImageJ (v2.0.0-rc-69/1.52i). A 3D rendering of vascular surfaces and microglia were generated using IMARIS (v9.8).

### Two-photon live imaging of embryonic LGE

Timed-pregnant mice (E12.5) were bred using female CD1 animals (CRL) and male *Cx3cr1*^*GFP*^ (B6.129P2(Cg)-*Cx3cr1*^*tm1Litt*^/J, Jax Stock no. 005582) to visualize macrophages using GFP and to maximize litter size. The dams were anesthetized with ketamine/xylazine (60–120 mg kg^−1^and 5–10 mg kg^−1^, respectively, intraperitoneal) and placed on a heating pad. A laparotomy was performed, and the embryos were gently exposed. Vasculature labeling was achieved by placental injection of ~7.5 μl of 25 mg ml^−1^Texas Red dextran (70 kD, Thermo Fisher Scientific). Following a 10 min wait to allow the dextran to circulate, the samples with the placenta attached were transferred to an imaging chamber filled with artificial cerebrospinal fluid at 37 °C (CSHL formulation: https://cshprotocols.cshlp.org/content/2011/9/pdb.rec065730.full, 119 mM NaCl, 2.5 mM KCl, 1 mM NaH_2_PO_4_, 26.2 mM NaHCO_3_, 1.3 mM MgCl_2_, 2.5 mM CaCl_2_ and modified glucose amount to 35 mM glucose). The developing skull and cerebral CTX were removed, exposing the lateral ventricle and LGE. Oxygenated (95:5 O_2_:CO_2_) and warmed artificial cerebrospinal fluid was continuously circulated in the imaging chamber.

Two-photon imaging of immune cells was performed using either resonant-scanning (512 × 512 pixels per frame, 8.1 frames per second) or Galvo-scanning (1,024 × 1,024 pixels per frame, 1.3 frames per second) with a two-photon microscope (Olympus MPE-RS Multiphoton Microscope) and a 25× objective (Olympus XLSLPLN25XSVMP2, 0.95 NA, 8 mm WD), varying from 1× to 3× zoom. The recordings were between 15 and 30 min in duration. Volume scanning was achieved using a piezoelectric scanner (nPFocus250). The laser power at 920 nm (Mai Tai DeepSee laser, Spectra Physics) measured below the objective was 30–70 mW. The resonant-scanning acquisitions were processed and registered using previously published code^53^. Galvo-scanning acquisitions were processed and registered using previously published Fiji registration plugins^54^.

### TEM and IEM

For TEM, mouse embryos were fixed in 3% glutaraldehyde and 1% paraformaldehyde in 0.1 M sodium cacodylate buffer (pH 7.4) overnight. Following fixation, the tissues were processed through 2% osmium tetroxide and 4% uranyl acetate, then dehydrated and embedded in Eponate 12 resin (Ted Pella). Ultrathin sections were sectioned at 70 nm thickness, collected on copper grids and imaged in a Phillips Tecnai10 transmission electron microscope using FEI software. For IBA1^+^ IEM, human prenatal brains at GW21 were fixed in 4% paraformaldehyde for 7 days and then were cut into 0.5 cm coronal blocks. After selecting the region of interest, 100 μm sections were obtained with a vibratome (Leica VT-1000 S). Preembedding immunogold staining was performed by incubating sections in 1:150 primary antibody rabbit anti-Iba1 (WAKO) and in goat anti-rabbit colloidal gold-conjugated secondary antibody (1:50; UltraSmall) as described previously^55^. Then the slice with the immunogold staining were post-fixed with 1% osmium tetroxide with 7% glucose for 30 min, rinsed, dehydrated and embedded in araldite (Durcupan). Semithin sections (1.5 μm) were cut with an Ultracut UC-6 (Leica), mounted on gelatin-coated slides and stained with 1% toluidine blue. These sections were examined under a light microscope (Eclipse E200, Nikon). To identify individual cell types, ultrathin sections (70 nm) were cut, stained with lead citrate (Reynolds solution) and examined under a transmission electron microscope (Tecnai Spirit G2, FEI). The images were acquired using Radius software (version 2.1) with a XAROSA digital camera (EMSIS GmbH).

### In vitro cultures, angiogenesis assays and live imaging

HUVEC (ATCC) were cultured at 37 °C and 5% CO_2_ in an Endothelial Cell Growth Medium-2 BulletKit (EGM-2, Lonza) with the following supplements: fetal bovine serum, human recombinant epidermal growth factor, hFGF-B, R3-IGF-1 (5 ng ml^−1^), VEGF (2 ng ml^−1^), hydrocortisone, ascorbic acid, GA-1000 and heparin. Only HUVEC between passages 2 and 6 were used in experiments. For in vitro angiogenesis assays, 96-well plates were coated with undiluted Matrigel (growth factor-reduced basement membrane matrix, Corning) and seeded with HUVEC in EGM-2. At the same time, 10,000 or 20,000 CD45^+^ cells were added to each well for evaluating the role of immune cells in angiogenesis. Same densities of CD45^−^ cells were used as controls. In experiments where CD45^+^ cells were labeled, the cells were transfected with AAV–CMV–GFP (Vector Biolabs) at 200 multiplicity of infection (MOI) and incubated at 37 °C for 30 min, before washing with PBS. In assays with recombinant proteins, the following concentrations were added: 25 ng ml^−1^VEGF (R&D Systems), 5 ng ml^−1^IGF-1 (PeproTech), 5 μg ml^−1^ELANE (R&D Systems)^56^, 20 μg ml^−1^AZU1 (R&D Systems) and 10 μg ml^−1^CXCL16 (R&D Systems)^57^. From 0 to 48 h, vascular tube formations were imaged every 5–10 min using a 4× objective on Incucyte S3 Live-Cell Analysis Systems (Essen BioScience).

### Microvessel fabrication, vascular permeability measurement and imaging

Human dermal microvascular endothelial cells (hMVEC-Ds, Lonza) were maintained in EGM-2-MV (microvascular) growth medium (Lonza) and used at passages 2–7. The cells were maintained at 37 °C in 5% CO_2_ in a humidified incubator. Cell-line authentication (performance, differentiation and STR profiling) was provided by Lonza. Mycoplasma testing was performed on cells using a Mycoplasma Polymerase Chain Reaction Detection Kit (Applied Biological Materials). Microvessels were fabricated as previously described^39^. Briefly, microfluidic devices were made using soft lithography. Polydimethylsiloxane (Sylgard 184, Dow-Corning) was mixed at a ratio of 10:1 (base:curing agent) and cured overnight at 60 °C on a silicon master. Polydimethylsiloxane was then cut from the silicon master, trimmed and surface-activated by plasma treatment for 30 s. The devices were then bonded to glass and treated with 0.01% poly-l-lysine for 2 h, washed three times with water, treated with 1% glutaraldehyde for 15 min, washed three times with water and sterilized with 70% ethanol for 1 h. Steel acupuncture needles (160 μm diameter, Tai Chi) were sonicated in 70% ethanol for 3 min and introduced into each sterile device. Assembled devices were dried in a vacuum desiccant chamber for at least 30 min. Collagen type I (Corning) solution in 10× Dulbecco’s modified Eagle medium and 10× reconstitution buffer (0.26 M NaHCO_3_ and 0.2 M HEPES buffer) was titrated to pH 8.0 with 1 M NaOH to make a final concentration of 2.8 mg ml^−1^collagen I. The collagen solution was then injected into the microfluidic devices and polymerized for 15 min at 37 °C. Sterile PBS was added to the devices overnight, then the needles were removed to create 160-μm-diameter channels in the collagen gel. Growth medium was then added to the devices for at least 30 min before cell seeding. hMVECs were collected with 0.05% trypsin–EDTA and centrifuged at 200*g*for 3 min. The cells were resuspended at 1 × 10^6^ cells ml^−1^in EGM-2-MV, 50 μl of cell suspension was introduced into the devices and cells were allowed to adhere to collagen for 15 min before washing with growth medium. The devices were cultured on a lab rocker (variable speed rocker, VWR) to induce oscillatory shear stress (2 r.p.m., 30° tilt angle). The medium was changed every 24 h. Two days after seeding, devices were given EGM-2-MV media, which contained either (1) 20 μg ml^−1^CXCL16 (R&D Systems) and 20 μg ml^−1^activated ELANE (R&D Systems) or (2) 100 μg ml^−1^AZU1 (R&D Systems).

Vascular permeability was quantified as previously described (Polachek et al., 2019). Briefly, 25 μg ml^−1^fluorescent dextran (fluorescein isothiocyanate dextran 70 kDa, Sigma-Aldrich) was introduced into the perfusion medium, and dextran diffusion was imaged in real time with a Yokogawa CSU-X1 inverted spinning-disk Nikon Ti-E confocal microscope with a 10× air objective, 488 nm solid-state laser and a high-resolution interline CCD (cMyo, Photometrics). A timelapse microscopy was then used to measure the flux of dextran into the collagen gel. Using MATLAB, the resulting diffusion profile was fitted to a dynamic mass-conservation equation, with the diffusive-permeability coefficient (*P*_D_) defined by *J* = *P*_D_(*c*_vessel_ − *c*_ECM_), where *J* is the mass flux of dextran, *c*_vessel_ is the concentration of dextran in the vessel and *c*_ECM_ is the concentration of dextran in the perivascular extracellular matrix (ECM).

The devices were fixed immediately after barrier function measurements with 4% paraformaldehyde in PBS with calcium and magnesium at 37 °C for 15 min. The devices were rinsed three times with PBS, and the fixative was quenched with 100 mM glycine for 30 min. The devices were permeabilized with 0.25% Triton X-100 in PBS for 10 min and blocked with 2% bovine serum albumin in PBS for 1 h. Primary and secondary antibodies were applied in 2% BSA in PBS for 1 h each and were rinsed with PBS for 30 min between each incubation. Immunostained devices were imaged using a 20× air objective on either a Yokogawa CSU-W1 SoRA spinning disk confocal on a Nikon Ti-2 microscope equipped with a sCMOS camera (ORCA-Fusion BT, Hamamatsu Photonics) or a CSU-10 spinning disk confocal on a Nikon Ti-E microscope equipped with a CoolSnap HQ2 cooled charge-coupled camera (Photometrics).

### FACS and high-dimensional flow cytometry

To isolate immune cells from prenatal human brain tissues, we adapted a previously published protocol^4^. Briefly, brain tissue was minced with a scalpel and digested with collagenase/dispase (3 mg ml^−1^, Sigma) for 30 min at 37 °C with rotation, triturated in 2% fetal bovine serum in PBS with DNase (0.25 mg ml^−1^) and centrifuged through 22% Percoll (Sigma) to remove debris. The cells were then stained with acridine orange/propidium iodide (AO/PI) cell viability dye and counted with the Cellaca MX High-throughput Automated Cell Counter (Nexelcom). Approximately 3 million cells were aliquoted from each sample into a 96-v-well plate (Corning) and incubated with Zombie Aqua Fixable Viability Dye (Thermo) for 20 min on ice and in the dark. After viability dye incubation, the cells were washed with sort buffer (PBS/2% FCS/2 mM EDTA) and incubated with Human Fcx (BioLegend) to block non-specific antibody binding. The cells were then washed with sort buffer and incubated with cell surface antibody mix diluted in Brilliant stain buffer (BD Biosciences) for 30 min on ice and in the dark. Following antibody stain, the cells were washed twice with sort buffer. The cells were then resuspended in fixation buffer (BD Bioscience) for 20 min on ice and in the dark. The flow cytometry data were acquired using FACS Diva software v.7 (BD), and the data obtained were analyzed using FlowJo v10.8.2 (LLC).

### In vitro angiogenesis assay and RNA-seq

Following Percoll centrifuge, the samples were incubated on ice for 15 min with PECy7-conjugated mouse anti-hCD45 (1:200, BD Biosciences). After washing, the samples were resuspended in HBSS buffer with DAPI (1:500, Thermo Fisher) to exclude dead cells. the cells were sorted using Becton Dickinson FACSAria with 13 psi pressure and 100 μm nozzle aperture. All FACS gates were set using unlabeled cells, single-color and isotype controls from human samples. FACS-processed cells were immediately used for scRNA-seq or angiogenesis assays. The FACS graphs were prepared using FlowJo (v10.6.2).

### Bulk RNA-seq, analysis and GSEA

For each sample, RNA was extracted from 1,000 cells and loaded as input for complementary DNA amplification. cDNA concentration was measured with NanoDrop and diluted to 0.4 ng μl^−1^. The ibraries were constructed using Takara SMART-seq (v4) and Illumina Nextera XT following the manufacturer’s instructions. The library pools were quality-controlled and normalized using Illumina MiniSeq before pair-ended (100 bp reads) sequencing with Illumina HiSeq4000. Finally, the reads were aligned to hg38 using STAR (v2.7.2.b). EdgeR (version 3.34.1) in R software was used to normalize raw counts and perform differential gene expression analyses. In addition, DESeq2 (version 1.32.0) was used to generate heat maps with variance stabilizing transformation. A GSEA was performed with biological pathways in GO (c5.go.bp) from MSigDB (https://www.gsea-msigdb.org/gsea/msigdb/).

### scRNA-seq

#### Sample preparation.

The cells after FACS were processed with single-cell 3′ v3 and v3.1 kits (10x Genomics), following the manufacturer’s instructions. In each sample, 10,000 cells were targeted for capture, and 12 cycles of thermal cycling were used for cDNA amplification and library amplification. The libraries were sequenced as per manufacturer recommendation on a NovaSeq S2 flow cell.

#### scRNA-seq data processing, UMAP visualization and clustering.

Gene counts were obtained by aligning reads to the hg38 genome (refdata-gex-GRCh38-2020- A) using CellRanger (v.3.1.0) (10x Genomics). Only high-quality cells were included for further analysis with the following criteria: number of genes per cell between 200 and 6,000, number of RNA molecules per cell less than 40,000 and mitochondrial RNAs per cell less than 5%. The doublets were checked with Scrublet, which predicted that minimal doublets were present in the dataset after filtering. After applying these quality control steps, the control samples yielded 56,314 cells, and the GMH samples yielded 17,187 cells. After filtering, the dataset was first batch corrected by sample using SCTransform from Seurat (version 4.1.0). A UMAP was then used to project the dataset into two-dimensional space with the top 30 principal components. Clustering was performed with the Louvain–Jaccard method, and top expressed genes in more than 50% of cells in each cluster were designated as marker genes.

#### scRNA-seq cell type annotation, differential gene expression and GO analysis.

Cell types were annotated based on UMAP reduction and unbiased gene marker analysis using Seurat’s FindAllMarkers function. Genes expressed in >50% of cells in each cluster with log fold change >1.52 were selected as cell type markers. For pseudobulk differential expression analysis between control CD45^+^ cells from the GE and the CTX, as well as between CD45^+^ cells from control and GMH cases, we utilized Seurat’s built-in DESeq2 function and filtered to show only DEGs with false discovery rate (FDR) <0.05 and fold change >1.2. For GO analysis, we used Metascape to perform statistical overrepresentation tests for DEGs in each condition. GO biological processes were chosen to represent enriched functional properties. The processes with FDR <0.05 were considered to be significant.

#### scRNA-seq integration with published scRNA-seq data.

Published or shared scRNA-seq data were projected onto UMAP plots of our dataset using ProjecTILs according to its instructions^58^. The mouse genes were converted to human ortholog genes using Ensembl accordingly.

#### scRNA-seq ligand–receptor matching (CellphoneDB and NicheNet).

For inputs into CellphoneDB (v2), normalized counts and their cell type information were exported from Seurat. Only ligands and receptors expressed in more than 25% of cells in each cluster were considered. A total of 3,000 cells were subsampled to produce the heat maps due to limited computational power, but they should accurately reflect the interaction frequencies between cell types. For NicheNet (v1.1.0)^33^, previously defined Seurat objects with their cell type information were used as inputs. Only ligands and receptors expressed in more than 20% of CD45^+^ cells or endothelial cells were considered. Only the top 200 differentially expressed target genes in >10% of the receiver cell type (that is, endothelial cells) were used for downstream analysis. Ligands from sender cells (that is, CD45^+^ cells) that were predicted to differentially regulate target gene expression in receiver cells were further filtered to only include those that were enriched in either the GE or in GMH cases. Both the width and the transparency of arrows in the circularized plots indicate interaction strengths between predicted ligand–receptor pairs.

#### Gene burden analyses.

Gene burden scores were calculated as the total number of DEGs normalized over the number of genes expressed. The DEGs were filtered to have fold change above 1.2 and FDR below 0.05. Each data point represents a pairwise comparison between a control sample and a GMH sample.

### Statistics and reproducibility

The data distribution was assumed to be normal, but this was not formally tested. Animals were randomly assigned to the experimental groups. For all statistical analysis, a *P* value less than 0.05 was considered significant. No statistical methods were used to predetermine sample sizes, but our sample sizes are similar to those reported in our previous publications^3,4^. No animals or data points were excluded from the analyses for any reason. Data collection and analysis were performed blinded to the conditions of the experiments and to the observers. The exact numbers of samples, including images and biological replicates, are indicated in the figure legends. Statistical analyses were done using Prism 10 (GraphPad). For comparisons between two groups, if normally distributed, two-tailed unpaired Student’s *t*-tests were performed.

### Software usage

The following software was used in this study: data collection: Leica TCS SP8, data analysis: Fiji (v2.0.0-rc-69/1.52i), IMARIS (v9.8), GraphPad Prism (v8.4.3), FlowJo (v10.6.2), R (v4.1.0), Rstudio (v1.1.463), CellphoneDB (v2), NicheNet (v1.1.0), DESeq2 (v1.32.0), STAR (v2.7.2.b), edgeR (v3.34.1), GSEA (v4.2.3), CellRanger (v.3.1.0), Seurat (v4.1.0) and ProjecTILs (v2.0).

## Extended Data

## Supplementary Material

Chen Supplementary Table 3

Chen_Supplementary_Table_4

Chen_Supplementary_Table_5

Chen_Supplementary_Table_6

Chen_Supplementary_Table_7

Chen_Supplementary_Video_2

Chen_Supplementary_Video_1

Chen_Supplementary_Video_3

Chen_Supplementary_Video_6

Chen_Supplementary_Video_4

Chen_Supplementary_Video_5

Chen_Supplementary_Video_7

Chen_Supplementary_Table_1

Chen_Supplementary_Table_2

## Data Availability

All RNA-seq data in this work are available through GEO (accession number PRJNA885959) and are publicly available as of the date of publication. Any FACS, flow cytometry, microscopy data, confocal or electron or other original data reported in this paper will be shared upon request.
