# Supplementary material for: Proinflammatory immune cells disrupt angiogenesis and promote germinal matrix hemorrhage in prenatal human brain
Source: Nat Neurosci. 2024 Sep 30;27(11):2115–29. doi: 10.1038/s41593-024-01769-2 (PMC11537974; doi:10.1038/s41593-024-01769-2)
Supplement: Supplementary file 1 [file 41593_2024_1769_MOESM1_ESM.pdf]

nature portfolio

Corresponding author(s): Eric J. Huang

Last updated by author(s): Jul 8, 2024

Reporting Summary

Nature Portfolio wishes to improve the reproducibility of the work that we publish. This form provides structure for consistency and transparency in reporting. For further information on Nature Portfolio policies, see our Editorial Policies and the Editorial Policy Checklist.

Statistics

For all statistical analyses, confirm that the following items are present in the figure legend, table legend, main text, or Methods section.

n/a	Confirmed	
☐	☒	The exact sample size (*n*) for each experimental group/condition, given as a discrete number and unit of measurement
☐	☒	A statement on whether measurements were taken from distinct samples or whether the same sample was measured repeatedly
☐	☒	The statistical test(s) used AND whether they are one- or two-sided<br>*Only common tests should be described solely by name; describe more complex techniques in the Methods section.*
☐	☒	A description of all covariates tested
☐	☒	A description of any assumptions or corrections, such as tests of normality and adjustment for multiple comparisons
☐	☒	A full description of the statistical parameters including central tendency (e.g. means) or other basic estimates (e.g. regression coefficient) AND variation (e.g. standard deviation) or associated estimates of uncertainty (e.g. confidence intervals)
☐	☒	For null hypothesis testing, the test statistic (e.g. *F*, *t*, *r*) with confidence intervals, effect sizes, degrees of freedom and *P* value noted<br>*Give P values as exact values whenever suitable.*
☒	☐	For Bayesian analysis, information on the choice of priors and Markov chain Monte Carlo settings
☒	☐	For hierarchical and complex designs, identification of the appropriate level for tests and full reporting of outcomes
☒	☐	Estimates of effect sizes (e.g. Cohen's *d*, Pearson's *r*), indicating how they were calculated

Our web collection on statistics for biologists contains articles on many of the points above.

Software and code

Policy information about availability of computer code

Data collection	Leica TCS SP8
Data analysis	Fiji (v2.0.0-rc-69/1.52i); IMARIS (v9.8); GraphPad Prism (v8.4.3); FlowJo (v10.6.2); R (v4.1.0); Rstudio (v1.1.463); CellphoneDB (v2); NicheNet (v1.1.0); DESeq2 (v1.32.0); STAR (v2.7.2.b); edgeR (v3.34.1); GSEA (v4.2.3); CellRanger (v.3.1.0); Seurat (v4.1.0); ProjecTILs (v2.0)

For manuscripts utilizing custom algorithms or software that are central to the research but not yet described in published literature, software must be made available to editors and reviewers. We strongly encourage code deposition in a community repository (e.g. GitHub). See the Nature Portfolio guidelines for submitting code & software for further information.

Data

Policy information about availability of data

All manuscripts must include a data availability statement. This statement should provide the following information, where applicable:

- Accession codes, unique identifiers, or web links for publicly available datasets
- A description of any restrictions on data availability
- For clinical datasets or third party data, please ensure that the statement adheres to our policy

Bulk and single cell RNA-sequencing (scRNA-seq) data for CD45+ cells from the prenatal brain in homeostasis and germinal matrix hemorrhage have been deposited to the SRA (accession #PRJNA885959). Source data for Figures 1-7 and Extended Data Figures 1-7 are available with the paper. All data supporting the findings of this study are available within the article supplemental materials.

Research involving human participants, their data, or biological material

Policy information about studies with human participants or human data. See also policy information about sex, gender (identity/presentation), and sexual orientation and race, ethnicity and racism.

Reporting on sex and gender	De-identified age-matched control cases (n=29) and cases with germinal matrix hemorrhage (n=16) of both sexes were collected from the Autopsy Service in the Department of Pathology at the University of California San Francisco (UCSF) and La Fe Biobank (see Supplementary Table S1 for details) with previous patient consent in strict observance of the legal and institutional ethical regulations.
Reporting on race, ethnicity, or other socially relevant groupings	The ethnicity of the samples in UCSF pediatric brain bank includes 71% Caucasian, 23% Hispanic, and 6% Asian. This demographic data reflect the racial makeup of the greater Bay Area in California.
Population characteristics	The ethnicity of the patients for UCSF Children's Hospital NICU includes Non-Hispanic White: 45.2%, Hispanic: 27.8%, Asian: 15.2%, Black: 9.4%, American Indian: 1.2%, and Others: 0.4%.
Recruitment	This study does not utilize recruitment plans to collect tissue samples.
Ethics oversight	The autopsy consent and all protocols for human prenatal brain tissue procurement were approved by the Human Gamete, Embryo and Stem Cell Research Committee (Institutional Review Board GESCR# 10-02693) at the University of California, San Francisco (UCSF) and by the UC San Diego Institutional Review Board (IRB 171379).

Note that full information on the approval of the study protocol must also be provided in the manuscript.

Field-specific reporting

Please select the one below that is the best fit for your research. If you are not sure, read the appropriate sections before making your selection.

☒ Life sciences ☐ Behavioural & social sciences ☐ Ecological, evolutionary & environmental sciences

For a reference copy of the document with all sections, see nature.com/documents/nr-reporting-summary-flat.pdf

Life sciences study design

All studies must disclose on these points even when the disclosure is negative.

Sample size	No power analyses were used to predetermine sample sizes. Sample sizes were determined based on the lab's previous experience working with similar clinical data and mouse models. The determination of sample size is further assisted by prior literature using similar experimental paradigms that yielded interpretable results.
Data exclusions	No data was excluded.
Replication	For quantifications of both human and mouse samples, each data point represents a biological replicate. For human umbilical vein endothelial cell (HUVEC) assays in Matrigel, at least 5 biological replicates were assessed in each age group or condition, with at least 3 technical replications for each biological sample. The exact biological replicates for each dataset are indicated in the figure legends and online-only METHODS.
Randomization	Mouse and human samples from both genders were used and all samples were randomly assigned to experimental groups. Experimental results were compared to both positive controls (when applicable) and with negative controls to determine background and specificity.
Blinding	All immunohistochemical and in situ hybridization data collection and analyses were performed by blinded observers. For scRNA-seq data analyses, experimenters were blinded to group allocation during data acquisition and analysis.

Reporting for specific materials, systems and methods

We require information from authors about some types of materials, experimental systems and methods used in many studies. Here, indicate whether each material, system or method listed is relevant to your study. If you are not sure if a list item applies to your research, read the appropriate section before selecting a response.

Materials & experimental systems

n/a	Involved in the study
☐	☒ Antibodies
☐	☒ Eukaryotic cell lines
☒	☐ Palaeontology and archaeology
☐	☒ Animals and other organisms
☒	☐ Clinical data
☒	☐ Dual use research of concern
☒	☐ Plants

Methods

n/a	Involved in the study
☒	☐ ChIP-seq
☐	☒ Flow cytometry
☒	☐ MRI-based neuroimaging

Antibodies

Antibodies used

Primary antibodies for IHC: Mouse anti-CD31 (DAKO, M082329-2, 1:200), Sheep anti-CD31 (R&D Systems, AF806, 1:250), Rabbit anti-IBA1 (FUJIFILM Wako Shibayagi, 019-19741, 1:3000), Goat anti-IBA1 (Novus Biological, NB100-1028, 1:250), Fluorescein labeled isolectin B4 (Vector Laboratories, FL-1201, 1:50), Rabbit anti-ZO-1 (Thermo Fisher Scientific, 40-2200, 1:100), Rat anti-BrdU (Abcam, ab6326, 1:500), Rabbit anti-S100A9 (Abcam, ab63818, 1:500), Mouse anti-HLA DR + DP + DQ (Abcam, ab7856, 1:200), Rabbit anti-ELANE (Abcam, ab131260, 1:1000), Mouse anti-CD16 (Santa Cruz Biotechnology, sc-20052, 1:100), Rat anti-CD68 (Bio-Rad, MCA1957, 1:3000), Goat anti-CXCL16 (Thermo Fisher Scientific, PA5-47977, 1:50), Mouse anti-VE cadherin (Santa Cruz Biotechnology, sc-9989, 1:300)

Secondary Antibodies for IHC: Donkey anti-mouse Alexa fluor 488 (Thermo Fisher Scientific, A-21202, 1:300), Donkey anti-sheep Alexa fluor 488 (Thermo Fisher Scientific, A-11015, 1:300), Donkey anti-mouse Alexa fluor 568 (Thermo Fisher Scientific, A-10037, 1:300), Donkey anti-rat Alexa fluor 594 (Thermo Fisher Scientific, A-21209, 1:300), Donkey anti-mouse Alexa fluor 647 (Thermo Fisher Scientific, A-31571, 1:300), Donkey anti-rabbit Alexa fluor 647 (Thermo Fisher Scientific, A-31573, 1:300), Donkey anti-goat Alexa fluor 647 (Thermo Fisher Scientific, A-21447, 1:300), Goat anti-Mouse Alexa fluor 647 (Thermo Fisher Scientific, A-21236, 1:300)

Conjugated Antibodies for Flow Cytometry: Mouse anti-CD45-PECy7 (BD Biosciences, 557748, 1:200), Mouse anti-CD11b-FITC (Thermo Fisher Scientific, 11-0112-41, 1:200), Mouse anti-CD16-PerCP Cy5.5 (BD Biosciences, 560717, 1:200), Mouse anti-CD14-APC (BD Biosciences, 561708, 1:200), Mouse anti-CD141(BDCA-3)-FITC (Miltenyi Biotec, 130-113-321, 1:40), Mouse anti-CD3-BB700 (BD Biosciences, 566575, 1:40), Mouse anti-CD11b(M1/70)-PE (Invitrogen, 12-0112-82, 1:40), Rat anti-CX3CR1-PE/Dazzle 594 (Biolegend, 341624, 1:20), Mouse anti-CD1c-PE/Cyanine7 (Biolegend, 331516, 1:40), Mouse anti-CD163-Alexa Fluor 647 (Biolegend, 333620, 1:40), Mouse anti-CD11c(3.9)-Alexa Fluor 700 (Invitrogen, 56-0116-42, 1:10), Mouse anti-CD45(HI30)-APC-eFluor 780 (Invitrogen, 47-0459-42, 1:40), Mouse anti-CD16-Brilliant Violet 421 (Biolegend, 302037, 1:40), Mouse anti-CD31-Brilliant Violet 605 (Biolegend, 303122, 1:40), Mouse anti-CD15(SSEA-1)-Brilliant Violet 650 (Biolegend, 323034, 1:40), Mouse anti-CD14-Brilliant Violet 711 (Biolegend, 301838, 1:40), Mouse anti-CD19-Brilliant Violet 785 (Biolegend, 302240, 1:40), Mouse anti-CD20-Brilliant Violet 785 (Biolegend, 302356, 1:40), Mouse anti-HLA-DR-BUV395 (BD Biosciences, 564040, 1:40), Mouse anti-CD64-BUV737 (BD Biosciences, 564425, 1:40), PECy7 Mouse IgG1, κ Isotype Control (BD Biosciences, 557872, 1:200), Alexa Fluor 488 Mouse IgG2a, κ Isotype Control (BD Biosciences, 557703, 1:200), PECy7 Mouse IgG1, κ Isotype Control (BD Biosciences, 347202, 1:200), APC Mouse IgG1, κ Isotype Control (BD Biosciences, 555751, 1:200)

Validation

Mouse anti-CD31 antibody (DAKO, M082329-2) was used to detect CD31 in human tissue (Georgopoulou et al., Nature Comm, 2021). Sheep anti-CD31 antibody (R&D Systems, AF806) was used to detect CD31 in human tissue (El Crosse et al., Cell Stem Cell, 2020, Campostrin et al., Nature Protocol, 2021). Rabbit anti-Iba1 antibody (Wako, 019-19741) was used to detect Iba1 in mouse brain (Marina OZ et al., J Neurosci, 2012, Stowell RD, et al., Nature, 2019). Goat anti-Iba1 antibody (Novus Biologicals, NB100-1028) was used to detect Iba1 in mouse cells (Imai Y & Kohsaka S, Glia, 2002). Fluorescein labeled isolectin B4 antibody (Vector Laboratories, FL-1201) was used to detect blood vessels in the mouse brain (Gogiraju et al., Scientific Reports, 2023). Rabbit anti-ZO-1 antibody (Thermo Fisher Scientific, 40-2200) was validated using siRNA mediated knockdown of target protein and using cell treatment by the manufacturer to ensure the specifity of antigen binding. Rat anti-BrdU antibody (Abcam, ab6326) was used to detect proliferating cells in the embryonic mouse brain (Muralidharan et al., J Neurosci, 2022). Rabbit anti-S100A9 antibody (Abcam, ab63818) was validated to detect S100A9 in human tissue by multiple publications listed in the vendor's website. Mouse anti-HLA DR + DP + DQ antibody (Abcam, ab7856) was validated in multiple publications listed on the manufacturer's website to detect HLA DR + DP + DQ in human tissues. Rabbit anti-ELANE antibody (Abcam, ab131260) was validated to detect ELANE in human tissue by the vendor. Mouse anti-CD16 antibody (Santa Cruz Biotechnology, sc-20052) was validated to detect CD16 in human tissue by the vendor. Rat anti-CD68 antibody (Bio-Rad, MCA1957) was used to detect CD68 in mouse brain tissue (Lopez et al., J Neurosci, 2011). Goat anti-CXCL16 antibody (Thermo Fisher Scientific, PA5-47977) was validated to detect CXCL16 in human tissue by the vendor. Mouse anti-VE cadherin antibody (Santa Cruz Biotechnology, sc-9989) was used to detect VE cadherin in human endothelial cells (Ayalon et al., J Cell Biol, 1994).
All conjugated antibodies used in flow cytometry from BD Biosciences (Mouse anti-CD45-PECy7 (557748), Mouse anti-CD16-PerCP Cy5.5 (560717), Mouse anti-CD14-APC (561708), Mouse anti-CD3-BB700 (566575), Mouse anti-HLA-DR-BUV395 (564040), Mouse anti-CD64-BUV737 (564425)) were validated with isotype controls using human immune cells by the vendor. All conjugated antibodies used in flow cytometry from Biolegend (Rat anti-CX3CR1-PE/Dazzle 594 (341624), Mouse anti-CD1c-PE/Cyanine7 (331516), Mouse anti-CD163-Alexa Fluor 647 (333620), Mouse anti-CD16-Brilliant Violet 421 (302037), Mouse anti-CD31-Brilliant Violet 605 (303122), Mouse anti-CD15(SSEA-1)-Brilliant Violet 650 (323034), Mouse anti-CD14-Brilliant Violet 711 (301838), Mouse anti-CD19-Brilliant Violet 785 (302240), Mouse anti-CD20-Brilliant Violet 785 (302356)) were validated with isotype controls using human immune cells by the vendor. Mouse anti-CD11b-FITC antibody (Thermo Fisher Scientific, 11-0112-41) was used to sort CD11b + human cells in flow cytometry (Lima et al., Nat Comm, 2021, Fond et al., JCI, 2015). Mouse anti-CD141(BDCA-3)-FITC antibody (Miltenyi Biotec, 130-113-321) was used to sort CD141+ human cells in flow cytometry (Chu et al., J Exp Med, 2012). Mouse anti-CD11b(M1/70)-PE antibody (Invitrogen, 12-0112-82) was used to sort CD11b+ human cells in flow cytometry (Miyamoto et al., Cell Reports, 2020, McKenzie et al., Cell Stem Cell, 2019). Mouse anti-CD11c(3.9)-Alexa Fluor 700 antibody (Invitrogen, 56-0116-42) was used to sort CD11c+ human cells in flow cytometry (Binnewies et al., Cell, 2019, Barry et al., Nat Med, 2018). Mouse anti-CD45(HI30)-

APC-eFluor 780 antibody (Invitrogen, 47-0459-42) was used to sort CD45+ human cells in flow cytometry (Combes et al., Nature, 2021, Mian et al., Nat Comm, 2015).

Eukaryotic cell lines

Policy information about cell lines and Sex and Gender in Research

Cell line source(s)	Human umbilical vein endothelial cells (ATCC, CRL-1730), Human neonatal dermal blood microvascular endothelial cells (Lonza, CC-2813).
Authentication	Human umbilical vein endothelial cells (ATCC, CRL-1730): Karyology performed for one batch of CRL-1730 in 1996 reflected a hypodiploid human cell line with a modal chromosome number of 45 occurring in 72% of the cells counted, all of which had monosomic N13. The rate of polyploid cells among this population was 15.8%. Cells express factor VIII.<br><br>Human neonatal dermal blood microvascular endothelial cells (Lonza, CC-2813): Dermal Microvascular Endothelial Cells are cultured to be ≥90% pure and specifically enriched for dermal LECs and BECs. Cryopreserved cells are shipped in third passage. Cryopreserved Dermal Microvascular Endothelial Cells are guaranteed through 12 population doublings, express CD31/105, von Williebrand Factor VIII, and are positive for acetyated low density lipoprotein uptake.
Mycoplasma contamination	All cell lines tested negative for mycoplasma contamination.
Commonly misidentified lines (See ICLAC register)	No commonly misidentified cell lines were used in this study.

Animals and other research organisms

Policy information about studies involving animals; ARRIVE guidelines recommended for reporting animal research, and Sex and Gender in Research

Laboratory animals	Mice carrying deletion of exon 5 of the mouse colony stimulating factor 1 receptor gene (Csf1r+/-) were obtained from the Jackson Laboratories (B6.Cg-Csf1rtm1.1Jwp/J, JAX #028064). Timed-pregnant mice (E12.5) were bred using 2-4 months old female CD1 animals (Charles River Laboratories) and male Cx3cr1GFP (B6.129P2(Cg)-Cx3cr1tm1Litt/J, JAX #005582) to visualize macrophages with GFP in two-photon live imaging. E12.5 Cdh5-Cre/+;S1pr1fl/fl mice and age-matched control embryos were provided by Dr. Julieta Alfonso (DKFZ, Germany). Mouse husbandry conditions, including ambient temperature, humidity and dark/light cycle followed the guidelines established by UCSF LARC.
Wild animals	No wild animals were used in the study.
Reporting on sex	All data on mice were collected with a balanced ratio of both sexes, since sex-based differences were not a focus of this study. For embryonic samples, sex was determined through genotype.
Field-collected samples	No field collected samples were used in the study.
Ethics oversight	All experiments were conducted in accordance with the University of California San Francisco Institutional Animal Care and Use Committee guidelines (IACUC Protocol #AN169548).

Note that full information on the approval of the study protocol must also be provided in the manuscript.

Plants

Seed stocks	n/a
Novel plant genotypes	n/a
Authentication	n/a

Flow Cytometry

Plots

Confirm that:

☒ The axis labels state the marker and fluorochrome used (e.g. CD4-FITC).

☒ The axis scales are clearly visible. Include numbers along axes only for bottom left plot of group (a 'group' is an analysis of identical markers).

☒ All plots are contour plots with outliers or pseudocolor plots.

☒ A numerical value for number of cells or percentage (with statistics) is provided.

Methodology

Sample preparation	Brain tissue was minced with a scalpel and digested with collagenase/dispase (3 mg/ml, Sigma) for 30 min at 37 °C with rotation, triturated in 2% FBS in PBS with DNase (0.25 mg/ml), and centrifuged through 22% Percoll (Sigma) to remove debris. Cells were then stained with AO/PI cell viability dye and counted with the Cellaca MX High-throughput Automated Cell Counter (Nexelcom). Approximately 3 million cells were aliquoted from each sample into a 96 v-well plate (Corning), and incubated with Zombie Aqua Fixable Viability Dye (Thermo) for 20 minutes on ice and in the dark. After viability dye incubation, cells were washed with sort buffer (PBS/2% FCS/2mM EDTA) and incubated with Human Fcx (Biolegend) to block non-specific antibody binding. Cells were then washed with sort buffer and incubated with cell surface antibody mix diluted in BV stain buffer (BD Biosciences) for 30 minutes on ice and in the dark. Following antibody stain, cells were washed twice with sort buffer. Cells were then resuspended in Fixation Buffer (BD Bioscience) for 20 minutes on ice and in the dark.
Instrument	BD FACSAria II, Fortessa.
Software	FACS Diva software v.7 (BD), FlowJo v10.8.2 (LLC).
Cell population abundance	Cell population abundance is indicated in Figure 5a.
Gating strategy	Figure 5a presents the gating strategy conducted for each sample. Cells were gated according to their FSC/SSC profile and live cells were gated according to their negative signal in the live dead channel.

☒ Tick this box to confirm that a figure exemplifying the gating strategy is provided in the Supplementary Information.
